# Index to Subjects

**Published:** 1933

**Authors:** 


### INDEX TO SUBJECTS

**Aachen,** E. P. Philpots, **3,** 104.

**Abdomen,** examination of, R. G. P. Lansdown, **36,** 33.
surgical physiology, T. S. Ellis, **8,** 98.

**Abdominal** cases, unusual, A. W. Prichard, **18,** 313.
conditions, surgical treatment, F. Fraser, **38,** 1.
fibroma simulating splenomegaly, D. A. Alexander, **26,** 60.
incisions, T. Carwardine, **18,** 204.
operations, J. Swain, **16,** 211 ; **18,** 103 ; **19,** 18, 121 ; **20,** 33.
section, J. R. Thomas, **15,** 145 ; J. L. Firth, **16,** 229.
surgery, C. A. Morton, **17,** 203 ; F. Lace, **36,** 1.
viscera, displacement of, T. Fisher, **19,** 210.
wall, fibroma of, J. L. Firth, **14,** 304.
*see also* Intestinal, Tuberculosis.

**Abortion,** incomplete, A. E. A. Lawrence, **6,** 8.
pathology of intrauterine death, W. D. Priestley, **6,** 55.
tubal, D. C. Rayner, **20,** 323.

**Abscess,** cerebellar, J. M. Clarke and C. A. Morton. **19,** 112.
cerebral, A. N. G. Gibbs, **2,** 121 ; *and see* Ear.
dental, grave cardiac symptoms due to, W. R. Ackland, **41,** 79.
with filariasis, A. L. Flemming, **24,** 139.
of liver, W. J. Fyffe, **2,** 245.
sub-diaphragmatic, J. Harper, **2,** 184.
of thigh from intestinal injury, G. H. Lilley, **4,** 181.

**Accident,** what is an ? C. Corfield, **31,** 148.

**Accommodation,** paralysis of, W. M. Beaumont, **20,** 213.

**Achademy,** *see* Elizabethes.

**Achondroplasia,** C. E. S. Flemming, **17,** 21 ; F. H. Edgeworth, **17,** 27.

**Acids,** dilute, in infection, I. W. Hall and A. D. Fraser, **38,** 158 ; *and see* Ferment.

**Acne** vulgaris, W. K. Wills, **23,** 113.

**Addison's** disease, J. E. Prichard, **3,** 269.
anæmia, C.N.S. in, G. Hadfield, **44,** 21.

**Adenoids,** *see* Tonsils, Tuberculosis.

**Adenoma,** renal, *see* Lindau.

**Adsorption** phenomena, O. C. M. Davis, **37,** 205.

**Adventure,** a surgical (autobiography), E. W. H. Groves, **50,** 1.

**Albuminuria,** *see* Eclampsia, Pregnancy.

**Alcohol,** use of, J. Dacre, **14,** 10 ; *see also* Heart.

**Ambulance,** 3rd Southern Midland Field, A. W. Prichard, **31,** 159.

**Amenorrhœa,** pigmentation in, A. E. A. Lawrence, **12,** 107 ; *and see* Gynæcology.

**Ameru,** female circumcision among, H. W. Brassington, **49,** 237.

**Anæmia,** dysphagia with, E. Watson-Williams, **49,** 209.
oxygen for, J. M. Clarke, **14,** 232.
pernicious, T. Cole, **4,** 231 ; J. M. Clarke, **31,** 97 ; G. L. Ormerod, **49,** 61.
*see also* Addison, Leukæmia.

**Anæsthesia,** C. H. Whiteford, **18,** 217 ; J. Freeman, **19,** 321 ; **27,** 238 ; A. L. Flemming, **44,** 1.
and surgery, A. L. Flemming, **48,** 233.
intratracheal, apparatus for, F. E. Shipway, **31,** 341 ; W. S. V. Stock and J. D. Fry, **31,** 344 ; E. W. H. Groves, **31,** 347.
spinal, A. S. Gubb, **22,** 231 ; W. J. Greer, **22,** 235 ; E. W. H. Groves, **25,** 305.
vomiting with, A. L. Flemming, **27,** 40.
*see also* Ether, Ethyl-Chloride, Medicine.

**Anæsthetics,** W. S. V. Stock, **46,** 197.
local, efficiency of, E. Watson-Williams, **42,** 164.

**Analgesia,** local, A. L. Flemming, **32,** 193.

**Anatomical** relations, effect of certain, J. Cantlie, **2,** 21.

**Aneurysm,** aortic, H. Waldo, **1,** 232 ; R. S. Smith, **4,** 233 ; H. F. A. Goodridge, **5,** 30 ; J. M. Clarke, **6,** 130 ; F. H. Edgeworth, **22,** 23.
paralysis of vocal cord by, B. J. Baron, **19,** 208.
simulation of, C. F. Coombs, **32,** 26.
orbital, A. W. Prichard, **5,** 267 ; **8,** 115.
*see also* Arteries.

**Angina** pectoris following sepsis, N. Neild, **44,** 263.

**Angioma** of cerebellum, *see* Lindau.
of liver, A. L. Sheppard, **25,** 46.

**Anthropology** old and new, Sir A. Keith, **47,** 287.

**Antibodies** in disease, F. Bushnell, **21,** 221 ; *and see* Tuberculin.

**Antrum,** maxillary, suppuration in, W. H. Harsant, **1,** 93 ; B. J. Baron, **15,** 13 ; *and see* Nasal.

**Anus,** artificial, following herniotomy, A. W. Prichard, **19,** 119.
and fæcal fistula, closure of, J. G. Smith, **13,** 1.

**Aorta,** aspiration of, *see* Pulmonary.
compression of, for post-partum hæmorrhage, F. P. Elliott, **25,** 121, 310.
rupture of, A. Prichard, **3,** 127 ; J. P. Bush, **3,** 129 ; H. Marshall, **3,** 178.
*see also* Aneurysm, Rheumatism.

**Aphonia,** functional, C. P. Crouch, **25,** 214 ; *and see* Hoarseness, Tuberculosis.

**Apoplexy**, prevention of, T. C. Allbutt, **23,** 1.
temperature in, J. M. Clarke, **17,** 97.

**Appendicitis**, J. G. Swayne, **4,** 108 ; J. Swain, **12,** 9 ; C. A. Morton, **18,** 30 ; **23,** 223, 360 ; T. Carwardine, **20,** 319 ; H. Chitty, **48,** 167.
cause of intestinal obstruction, R. Warren, **42,** 23.
extraperitoneal suppuration in, J. L. Firth, **14,** 36.
chronic and dyspepsia, H. Goodwyn, **35,** 18.
*see also* Cancer.

**Army-surgeon**, reminiscences, W. J. Fyffe, **7,** 145.

**Arrhythmia**, *see* Heart.

**Arterial** changes, senile, in child, F. W. T. Hughes and C. B. Perry, **46,** 219.
tension, high, C. F. Coombs, **46,** 35 ; *and see* Blood.

**Arteriosclerosis** and hyperpiesis, C. W. J. Brasher, **32,** 41.

**Arteries**, ligature of, J. P. Bush, **2,** 110.

**Artery**, *see* Aneurysm, Sympathectomy, Thrombosis, Vessel.
meningeal, rupture of, G. M. Smith, **26,** 58.

**Arthritis**, *see* Joint. Osteo-, Rheumatoid.

**Ascites**, *see* Thrombosis.

**Asthenopia**, *see* Eye.

**Asthma** (from goitre), J. R. Charles, **42,** 84.
surgical, W. J. Penny, **6,** 41.

**Ataxia**, J. M. Fortescue-Brickdale, **31,** 235.
in child, E. C. Williams, **29,** 163.
locomotor, F. H. Edgeworth, **23,** 234.
with Charcot's joint, H. Davy and A. G. B. Blomfield, **10,** 249.
tabetic, treatment, M. Faure, **24,** 210 ; **27,** 312.

**Atrophy**, *see* Liver, Muscular.

**Aural**, *see* Ear, Influenza.

**Auricles**, *see* Heart.

**Autonomic**, *see* Nervous.

**Axilla**, presentation of, F. P. Elliott, **19,** 130.

**Bacillus** of rheumatoid arthritis, A. S. Wohlmann, **14,** 123.
coli infection of urinary tract, J. R. Charles, **28,** 23.
in children, R. G. Gordon, **32,** 286.
in women, H. J. D. Smythe, **50,** 243.
typhoid fever with, J. I. Noble, **47,** 323.
Friedländer's, *see* Pulmonary.
Klebs-Lœffler, *see* Diphtheria.
pyocyaneus, *see* Septicæmia.
Y., *see* Dysentery.

**Bacteriology** of suppurations complicating pulmonary disease, J. O. Symes, **17,** 30.

**Baillie,** Miss A. B., resignation of, **40,** 167.

**Baldness,** H. Waldo, **23,** 107.

**Banti's** disease, *see* Splenomegaly.

**Barber-Surgeons,** the, G. Parker, **30,** 327.

**Baron,** B. J., Lord Mayor, **33,** 218.

**Bath,** A. B. Brabazon, **10,** 254 ; *see also* British, Rheumatic.
medicine during earlier years of, J. Wigmore, **31,** 193.

**Beddoe** Memorial Lecture 1930, Anthropology, Sir A. Keith, **47,** 287.

**Bell,** Dr. J. H., case of, **36,** 19, 89.

**Benzene** derivatives, relationship between toxicity and chemistry, O. C. M. Davis, **30,** 237.

**Bile-duct,** congenital obstruction, J. A. Birrell and A. W. Adams, **47,** 215.

**Biliary** cirrhosis, *see* Splenomegaly.
and intestinal sand, G. Parker, **28,** 112.

**Blackwater** fever, E. C. Williams, **22,** 128 ; J. M. Fortescue-Brickdale, **22,** 130.

**Blood** changes, *see* Anæmia, Cancer (sarcoma), Erythræmia, Hæmophilia, Leukæmia, Lymphocytosis.
counts, W. Gordon, **21,** 234.
poisoning in brothers, F. H. Edgeworth, **13,** 107.
pressure, standard method for, G. A. Stephens, **50,** 121.
stains in tropics, F. S. Smith, **34,** 82.

**Body,** cells and liquids of, G. A. Buckmaster, **44,** 154.
fluids, reaction of, to indicators, G. A. Buckmaster, **40,** 175.

**Bone** injuries, repair of, E. W. H. Groves, **38,** 142.
*see* also Cancer, Fracture, Mobility, War.

**Boswell's** *Life of Johnson*, B. M. H. Rogers, **28,** 289 ; **29,** 125.

**Bournemouth,** A. J. H. Crespi, **7,** 36.

**Boys,** physique of, B. M. H. Rogers, **27,** 27.

**Brachial,** *see* Neuritis.

**Bradycardia,** R. G. Fendick and G. Parker, **17,** 113.
eye symptoms in, F. R. Cross, *ibid.*

**Brain,** *see* Abscess, Cancer, Tumour.
of lioness, W. B. Kesteven, **3,** 246.

**Branchial** cleft, dermoid cyst of, T. Carwardine, **14,** 145.
fistula, G. Parker, **2,** 89.

**Breast,** diagnosis of swelling, C. A. Morton, **30,** 121 ; *and see* Cancer.

**Bright,** Richard, **50,** 170; commemoration, **44,** 221.

**Bristol,** cholera in, *see* Symonds.
crematorium for, **42,** 55.
health of, D. S. Davies, **22,** 55, 382; **24,** 137; **25,** 336.
Hotwells, L. M. Griffiths, **20,** 1, 142, 193.
early medical teaching in, F. R. Cross, **44,** 73.
medical, in seventeenth century, G. Parker, **29,** 201.
in eighteenth century, W. H. Harsant, **17,** 297.
Medical Dramatic Club, **47,** 88.
Medical Reading Society, L. M. Griffiths, **25,** 222.
M.O.H., **39,** 69.
Medical School, A. Prichard, **10,** 264.
address to students, J. Beddoe, **1,** 235; E. Fry, **11,** 267.
centenary, **46,** 232; **50,** 134, 183.
dinner, **42,** 241; **49,** 325.
history of (review), **50,** 199; *and see* History, University.
medical societies, G. M. Smith, **32,** 268.
*Medico-Chirurgical Journal,* history of, P. Watson-Williams, **50,** 151.
Medico-Chirurgical Society—
dinner, **44,** 233.
foundation, G. Parker, **33,** 129.
laws, **26,** 370.
milk week, **41,** 41.
open-air Hospital School, **42,** 53.
Royal Visit, **42,** 138, 190.
*see also* British, Clifton, Elizabethes, Encephalitis, Hospital, Infirmary, Library, Meningitis, Plague, Tuberculosis, Small-pox, University, War.

**Bristol's** contributions to medical progress, **50,** 165.

**British Association,** Bristol meeting, **47,** 248.

**British Medical Association,** Bath, **42,** 190; Bristol, **12,** 161; *and see* **50,** 181; Cheltenham, **19,** 276.

**British Medical Library Association,** **27,** 95.

**Bromoform** poisoning, R. Waterhouse, **18,** 129.

**Bronchi,** rhythmical contractions of, P. Watson-Williams, **21,** 6.

**Bronchitis,** F. H. Edgeworth, **17,** 197.
diplococcal, P. Watson-Williams, **20,** 135.

**Broncho-pneumonia,** *see* Influenza.

**Bronchoscopy,** *see* Laryngoscopy.

**Brown,** John, centenary, **28,** 265.

**Bullets,** *see* War.

**Burden** mental research trust, **50,** 58.

**Buxton,** A. J. H. Crespi, **9,** 167.

**Cæcum,** *see* Cancer.

**Cæsarean** section, W. C. Swayne, **22,** 120.

**Calculus,** renal, E. J. T. Jones, **21,** 245.
vesical, C. F. Pickering, **4,** 183 ; J. G. Smith, **5,** 177 ; on foreign body, H. Benham and J. G. Smith, **4,** 38.
in typhoid carrier, D. Wood, **37,** 148.
urethral, C. A. Morton, **28,** 127.
urinary, C. A. Moore, **31,** 126 ; *see also* X-rays.

**Calmette's** reaction, *see* Tuberculosis.

**Canada,** President in, **44,** 274.

**Cancer,** cardiac sign in, W. Gordon, **32,** 19.
chemotherapy, J. A. Murray, **45,** 217.
diagnosis and treatment, N. C. Dobson, **7,** 225 ; A. T. Todd, **44,** 144 ; C. A. Joll, **50,** 201 ; *and see* Radium, Radiotherapy, Tumour.
surgery of, C. A. Morton, **29,** 289 ; G. Turner, **44,** 141.
of breast, A. R. Short, **41,** 64 ; (operation), C. A. Morton, **12,** 25 ; **20,** 305 ; C. H. Whiteford, **18,** 336 ; *and see* Breast.
of cæcum, diagnosis from appendicitis, C. A. Morton, **39,** 82.
within duodenal loop, H. B. Murgoci, **44,** 195.
of jaw, J. G. Smith, **1,** 128.
of larynx, B. J. Baron, **7,** 78 ; *and see* Hoarseness.
of œsophagus (gastrostomy), J. G. Smith, **1,** 122 ; *and see* Deglutition, Dysphagia.
of pancreas, J. Swain, **26,** 45.
of pylorus (gastrojejunostomy), J. P. Bush, **20,** 57.
of stomach, J. M. Clarke, **12,** 181 ; J. M. Fortescue-Brickdale, **31,** 108.
of tongue, B. J. Baron, **8,** 80 ; C. H. Whiteford, **24,** 45.
of tonsil, E. Watson-Williams, **47,** 78 ; *and see below* (Sarcoma).
of uterus, J. G. Swayne, **8,** 119 ; W. H. C. Newnham, **15,** 24 ; E. W. H. Groves, **21,** 128 ; (endometriomata), A. D. Fraser, **44,** 113 ; varieties of, W. R. Williams, **17,** 314.
rodent ulcer, *see* Lupus.
sarcoma, blood changes in, F. G. Bushnell, **25,** 321.
spontaneous disappearance, G. M. Smith, **18,** 30.
of bone, preservation of limb, C. A. Morton, **24,** 308.
of brain (shoulder-centre), J. P. Bush and J. E. Shaw, **13,** 99.
of intestine, T. Fisher, **19,** 29.
of palate, W. J. Greer, **18,** 211 ; and tonsil, P. Watson-Williams, **37,** 51.
compression of spinal cord, E. L. Fox, **1,** 100.
of spleen, A. L. Taylor, **46,** 12.

**Cardiac, Carditis,** *see* Heart.

**Cardiograph** (sphygmograph), G. M. Smith, **1,** 71.

**Carpus**, dislocation of, T. Mitchell, **17**, 121.
**Carrel-Dakin**, *see* War.
**Carrick** and Bristol Medical Library, **30**, 174.
**Caruncle**, *see* Urethra.
**Cataphoresis**, *see* Ionization.
**Cat-bite**, W. J. Penny, **6**, 42.
**Catarrh**, *see* Nasal.
**Catheterization**, *see* Cystoscopy.
**Cattle**, *see* Tuberculin.
**Cauda** equina, lesion of, J. E. Shaw and J. P. Bush, **11**, 161.
**Cells**, *see* Body.
**Centroscope**, *see* X-ray.
**Cerebellum**, *see* Abscess, Lindau, Tumour.
**Cerebral**, *see* Abscess, Cortex, Heart, Paralysis, Pneumonia, Pregnancy, Vessels.
**Cerebro-spinal**, *see* Meningitis, Syphilis.
fluid, chloride content, H. Rogers, **45**, 197.
**Cervical** erosion, G. Hadfield, **43**, 151 ; H. J. D. Smythe, **43**, 156.
rib, R. Waterhouse, **31**, 232.
vertebræ, inflammation round, J. K. Spender, **11**, 1.
*see also* Cyst.
**Chancre**, *see* Syphilis.
**Charcot**, *see* Ataxia.
**Charities**, abuse of, E. Sturge, **22**, 273.
**Cheltenham**, J. H. Garrett, **11**, 241 ; *and see* (B.M.A.), **19**, 276.
**Chemistry**, relation to medicine, F. Francis, **40**, 119 ; *and see* Benzene, Pharmacy.
**Chemotherapeutics**, M. Nierenstein, **36**, 151 ; (cancer), J. A. Murray, **45**, 217.
**Children**, E. L. W. Dunbar, **8**, 192 ; *and see* Arterial, B. Coli, Heart, Infant, Leukæmia, Tuberculosis, Vomiting.
guidance, Mrs. Posthuma, **47**, 111 ; R. J. A. Berry, **47**, 120 ; R. G. Gordon, **47**, 126.
**Chloral** hydrate, J. K. Spender, **7**, 1.
**Chloroform**, *see* Ether.
**Cholecystotomy**, T. C. Grey, **16**, 311 ; A. R. Short, **31**, 34 ; *and see* Jaundice.
**Cholelithiasis**, J. Swain, **23**, 208 ; T. Carwardine, **26**, 317 ; E. Stack, **30**, 49 ; *and see* Intestinal obstruction.
**Cholera** in Bristol, **50**, 57.
**Chorea**, E. L. Fox, **2**, 73 ; J. M. Clarke, **6**, 131 ; C. F. Coombs, **29**, 51.
and rheumatism, J. J. S. Lucas, **22**, 239.

**Circumcision,** *see* Ameru.

**Cirrhosis,** biliary, *see* Splenomegaly.

**Cleft-palate,** E. Fawcett, **24,** 237.

**Clifton,** J. Beddoe, **7,** 114 ; *and see* Suicide, Zoological.

**Club-foot,** W. H. Harsant, **1,** 97 ; A. W. Adams, **43,** 232 ; *and see* Deformity.

**Cocaine,** **2,** 280 ; *and see* Anæsthesia, etc.
injurious effects of, F. R. Cross, **4,** 128.

**Colitis,** case resembling mucous, E. G. Trevithick, **16,** 120.
polyposa, C. F. Coombs, **24,** 31 ; *see also* Pericolitis.

**Coliuria,** *see* Bacillus Coli.

**Collargol,** J. M. Fortescue-Brickdale, **21,** 337.

**Colliery** explosion, cause of death in, T. Mitchell, **15,** 235.

**Colloidal** state of matter, F. Francis, **45,** 177.

**Colon,** redundant, G. B. Bush, **45,** 181 ; *see also* Diverticulitis, Tuberculosis.

**Colour-vision,** test for, **28,** 175.

**Colston** Research Society, **44,** 139 ; dinner, **45,** 152.

**Conditioned** response, C. Lloyd Morgan, **41,** 166.

**Consciousness,** double, F. H. Edgeworth, **25,** 186.

**Constipation,** F. H. Edgeworth, **31,** 40 ; *and see* Fæces.

**Consumption, Contagion,** *see* Infection, Tuberculosis.

**Continuity** and change in life, T. Carwardine, **42,** 201.

**Convulsions,** *see* Eclampsia.

**Coombs,** Carey F., memorial, **50,** 134.

**Cord,** *see* Delivery.

**Coronary** obstruction, C. F. Coombs, **44,** 249 ; G. Hadfield, **44,** 257.
thrombosis, C. F. Coombs, **49,** 277.

**Cortex :** head injuries illustrating function, R. G. Gordon, **40,** 139.

**Cotton-seed,** *see* Dermatitis.

**Craniotomy** to be abolished ? J. G. Swayne, **5,** 1 ; *and see* Epilepsy.

**Cremation,** J. G. Davey, **3,** 81 ; *and see* Bristol.

**Cretinism** and puerperium, D. A. Alexander, **27,** 128.

**Croup,** bronchial, G. A. Brown, **4,** 115 ; *see also* Bronchitis, Diphtheria.
non-bacillary, J. O. Symes, **18,** 25.

**Curriculum,** *see* Specialism.

**Cutaneous** eruption, *see* Dermatitis, Diphtheria.

**Cyanosis,** *see* Influenza.

**Cyst,** branchial dermoid, T. Carwardine, **14,** 145.
mesenteric, A. W. Prichard, **22,** 332.
in neck, C. A. Morton, **29,** 160.
pancreatic, T. Carwardine, **26,** 319 ; *and see* Lindau.
tubo-ovarian, J. L. Firth, **16,** 229.
unusual, C. A. Morton, **12,** 232 ; *and see* Neuroma.

**Cystocele,** *see* Prolapse.

**Cystoscopy** and urethral catheterization, T. Carwardine, **19,** 303.

**Cystotomy,** *see* Tumour.

**Davies,** Dr. D. S., presentation, **45,** 151.

**Dawson** of Penn, Lord, **37,** 187.

**Deafness,** A. J. M. Wright, **49,** 256 ; *and see* Ear.

**Death,** certificate of cause, A. Prichard, **6,** 153 ; *and see* Colliery, Pneumonia, Tuberculosis.

**Deformity,** congenital, of hand and foot, J. G. Smith, **1,** 130.
*see also* Club-foot, Ear, Intestinal, Nasal, Surgery.

**Deglutition,** disorders of, J. P. I. Harty, **40,** 195 ; *and see* Dysphagia.

**Delivery,** the hour of, J. G. Swayne, **6,** 174 ; *see also* Craniotomy, Ergot, Hæmorrhage, Labour, Pregnancy, Presentation, Post-partum, Symphysiotomy, Tumour.
forceps, J. G. Swayne, **10,** 153.
compression of cord, J. G. Swayne, **11,** 229.

**Delusions,** reality of, H. Devine, **45,** 19.

**Dental** infections, G. F. Fawn, **47,** 137 ; R. H. McKeag, **47,** 143 ; *see also* Abscess, Neuralgia, Oral.

**Dermatitis** from without and dermatitis from within, A. J. Harrison, **24,** 325 ; *see also* Diphtheria.
cotton-seed, and pediculoides ventricosus, J. A. Nixon, **33,** 73.

**Dermatology,** problems of, W. K. Wills ; *see also* Acne, Lupus, Progress, Radiotherapy, Sclerodermia, Urticaria, Warts.

**Dermoid,** *see* Cyst.

**Development** impeded by defective nasal respiration, P. Watson-Williams, **24,** 17.

**Diabetes** mellitus, gangrene in, C. A. Morton, **28,** 311.
insulin for, A. T. Todd, **40,** 182 ; in surgery, J. A. Nixon, **43,** 199.
secretin for, **24,** 218.
*see also* Sugar.

**Diet,** G. M. Smith, **7,** 73; *and see* Food, Infant, Milk, War.
of sick, R. S. Smith, **4,** 55.
and hygiene at North Pole (review), C. Clarke, **40,** 52.
problems of living at high altitudes, Mount Everest, T. Somervell, **40,** 170.

**Digitalis,** *see* Heart.

**Dilatation,** *see* Stomach.

**Diphtheria,** R. S. Smith, **2,** 61.
bacterioscopic diagnosis, F. W. Stoddart, **13,** 18.
isolation after, B. M. H. Rogers, **16,** 101.
paralysis following, A. S. Currie, **2,** 186.
and plague, D. S. Davies, **19,** 367.
virulent, T. Aubrey and R. B. Britton, **46,** 141.
cutaneous eruption with Klebs-Lœffler bacillus, W. K. Wills, **28,** 231.

**Diseases** bearing names of Saints, R. Fletcher, **30,** 295.

**Disinfection,** D. S. Davies, **6,** 83; *and see* Soap.

**Dislocation** of hand and carpus, T. Mitchell, **17,** 121.
of ulna and radius, R. J. H. Scott, **4,** 36.
*see also* Mobility.

**Disseminated** sclerosis, J. M. Clarke, **7,** 265.

**Diverticulitis** of pelvic colon, Sir C. Gordon-Watson, **41,** 112; *see also* Pelvic.
and portal pyæmia, F. H. Bodman and A. L. Taylor, **46,** 131.

**Doctor** as an expert and pioneer, C. J. Whitby, **24,** 222.
The Family, J. Dacre, **23,** 289.

**Dover,** Thomas, J. A. Nixon, **27,** 31; *also* **50,** 166.

**Drainage** continu par le gaz oxygène, Dr. Renoirdre, **33,** 39.

**Dreams,** G. H. Savage, **30,** 156.

**Droitwich,** A. J. H. Crespi, **6,** 1.

**Dropsy,** *see* Famine.

**Duboisia,** poisoning by, F. R. Cross, **4,** 127.

**Duodenum,** ulcer of, T. Carwardine, **41,** 16; **45,** 279; *see also* Cancer, Peptic, Stomach.
medical treatment, R. C. Clarke, **45,** 289.
perforated, G. M. Smith, **21,** 218.

**Dysentery** due to Y. Bacillus, R. H. Norgate and I. W. Hall, **33,** 44.

**Dysmenorrhœa,** R. S. S. Statham, **44,** 187.

**Dyspepsia,** A. T. Todd, **47,** 83; *and see* Appendicitis, Peptic, Stomach.
diagnosis, A. R. Short, **42,** 88.

**Dysphagia,** *see* Anæmia, Deglutition.

**Dyspnœa,** *see* Asthma, Goitre.

**Ear,** external; congenital abnormalities, E. Watson-Williams, **48,** 273.
middle, suppuration, W. H. Harsant, **5,** 37; C. F. Pickering, **7,** 32.
operative technique, P. Watson-Williams, **27,** 224.
intracranial complications, J. M. Clarke and J. L. Firth, **30,** 97.
sequelæ, F. H. Edgeworth, **8,** 18, 87.
nose and throat disease, P. Watson-Williams, **26,** 1; *see also* Influenza.
specialist's experiences at the Base, J. P. I. Harty, **34,** 39.
power, method of expressing, R. A. Chudleigh, **3,** 159.
*see also* Deafness, History, Progress, Vertigo.

**East,** visit to Near, G. Parker, **44,** 173; *and see* Egypt, Medicine, War.

**Eclampsia,** G. H. Temple, **47,** 307.
puerperal, J. G. Swayne, **9,** 1, 106; E. L. W. Dunbar, **24,** 132.
venesection in, J. Fuller, **1,** 111.

**Ectopic,** *see* Pregnancy (extra-uterine).

**Edridge-Green,** *see* Vision.

**Education,** medical, W. C. Swayne, **30,** 316; C. E. S. Flemming, **49,** 199; *and see* Bristol, Elizabethes, Specialism.
postgraduate, **44,** 275; J. M. Rattray, **28,** 193; J. M. Fortescue-Brickdale, **36,** 48; J. O. Symes, **42,** 1.

**Egypt,** P. Watson-Williams, **13,** 263; *and see* War.

**Electrical** department, cases from, G. Parker, **22,** 112.
reactions, *see* Paralysis.

**Electricity** in general practice, H. P. Tayler, **27,** 145.
uses of static, P. King, **24,** 233.

**Elizabethes** Achademy, Queene, L. M. Griffiths, **37,** 1.

**Embolism,** fat, A. W. Prichard, **2,** 54; *and see* Thrombosis.

**Empyema,** treatment, W. H. C. Newnham, **4,** 225; J. A. Nixon, **38,** 82; (Estlander's operation), H. S. Ballance, **25,** 285.
double, W. J. Fyffe, **11,** 233.

**Encephalitis** lethargica in Bristol, D. S. Davies *et al.*, **37,** 25.
ocular manifestations, A. M. Critchley, **45,** 113.

**Endocarditis,** *see* Heart.

**Endocrine** glands, *see* Gynæcology.

**Endometriomata,** A. D. Fraser, **44,** 113; *and see* Uterus.

**Enteric,** *see* Typhoid.

**Enteritis,** membranous, C. P. Crouch, **13,** 14.

**Epilepsy,** B. B. Fox, **4,** 172 ; J. V. Blachford, **47,** 169.
hystero-epilepsy, B. B. Fox, **14,** 224.
Jacksonian, craniotomy, W. J. Greer, **19,** 42.
sulphonal in, G. A. Ballantyne, **9,** 261.

**Episcope,** *see* X-rays.

**Epithelioma,** *see* Cancer.

**Ergot,** J. G. Swayne, **12,** 225.
variations in, J. M. Fortescue-Brickdale, **31,** 279.

**Erosion,** *see* Uterus.

**Erythræmia** or splenic polycythæmia, G. Parker, **37,** 91.

**Erythema** nodosum, pathology, A. C. Fisher, **45,** 137 ; *and see* Tuberculosis.

**Estlander,** *see* Empyema.

**Ether,** J. Freeman, **13,** 191 ; A. L. Flemming, **40,** 88 ; *see also* Anæsthesia.
or chloroform ? W. H. Harsant, **9,** 145 ; J. Freeman, **14,** 137.
nitrous oxide before, B. M. H. Rogers, **13,** 10.

**Ethyl** chloride, F. H. Rose, **20,** 53 ; C. H. Whiteford, **22,** 49 ; A. L. Flemming, **22,** 228.

**Everest,** Mount, *see* Diet.

**Exophthalmic,** *see* Goitre.

**Exostosis,** multiple, E. D. MacLean, **8,** 217.
subungual, W. R. Williams, **22,** 17.

**Explosion,** *see* Colliery.

**Extremities,** tonic contraction of lower, W. H. Webb, **4,** 42.

**Eye** disease, E. R. Chambers, **49,** 131 ; *see also* Encephalitis, Glaucoma, Heart, Iritis, Ophthalmia, Paralysis, Papillœdema, Ptosis, Rheumatoid, Retinoscopy, Tuberculosis, Vision.
and quackery, C. H. Walker, **38,** 129.
gunshot injury, E. H. E. Stack, **33,** 198.
headache, asthenopia and, F. R. Cross, **2,** 73.
metastatic inflammation, J. H. Parsons, **27,** 1.
signs of migraine, E. R. Chambers, **43,** 29.
-lid, upper, surgery, F. R. Cross, **4,** 186.

**Facial,** *see* Neuralgia, Paralysis.

**Fæcal** fistula, *see* Anus.

**Fæces,** retention of, J. E. Shaw, **3,** 43 ; *and see* Constipation, Intestinal, Stools.

**Failure,** the teachings of, E. M. Skerritt, **10,** 229.

**Famine-dropsy** as a food deficiency disease, J. A. Nixon, **37,** 137.

**Fatigue,** A. F. S. Kent, **35,** 47.

**Fawcett,** E., elected F.R.S., **40,** 167.

**Femoral,** *see* Thrombosis.

**Femur,** *see* Fracture.

**Ferments,** lactic acid, value of, I. W. Hall and W. A. Smith, **26,** 56.
protective, I. W. Hall, **31,** 304.

**Fibroid,** *see* Uterus.

**Fibroma,** *see* Abdominal.

**Filariasis,** J. P. Bush and J. A. Nixon, **25,** 218.
with abscess, A. L. Flemming, **26,** 139.

**Fingers,** wounds of, J. K. Spender, **3,** 73.

**Finsen** light, *see* Lupus.

**Fistula,** *see* Anus.

**Flat-foot,** A. Ogston, **2,** 1.

**Fletcher,** R., **28,** 172, 190, 367 ; **50,** 177.

**Fluids,** *see* Body.

**Focal** infection, *see* Eye, Nasal, Oral.

**Food,** influence of, on production and prevention of disease, J. A. Nixon, **47,** 255 ; *and see* Diet, Famine, Milk, Scurvy.
peptonized, W. H. Spencer, **2,** 32.

**Foramina,** *see* Skull.

**Forbes Fraser** Hospital, **40,** 154.

**Forceps,** *see* Delivery.

**Foreign** body, *see* Calculus, Gullet, War, X-ray.

**Forensic,** *see* Blood-stains.

**Fox,** E. Long, Lectures, *see* page 9.

**Fracture,** medullary bone-pegging in, C. F. Walters, **32,** 139.
ununited, E. W. H. Groves, **36,** 132.
*see* also Bone, Mobility, Olecranon, X-rays.
of femur, C. F. Pickering, **3,** 48.
of frontal bone, A. W. Prichard, **15,** 27.
of patella, A. W. Prichard, **17,** 104.

**France,** *see* War.

**Free,** John, **25,** 276 ; **50,** 165.

**Frenchay,** *see* Sanatorium.

**Friedländer's** bacillus, *see* Pulmonary.

**Fright,** effects of, J. R. Charles, **24,** 23.

**Frontal,** *see* Fracture, Sinus.

**Furunculosis,** L. S. Smith, **29,** 157.

**Gall-bladder** disease, E. Stack, **30,** 49 ; *and see* Cholecystotomy, Cholelithiasis, Intestinal.

**Gangrene,** *see* Diabetes, Thrombosis.

**Gasserian** ganglion, *see* Neuralgia.

**Gastric,** *see* Peptic, Stomach.

**Gastro-enterostomy,** T. Carwardine, **27,** 107 ; *and see* Cancer.

**Gestation,** *see* Pregnancy.

**Glaucoma,** F. R. Cross, **2,** 145.

**Glycosuria,** *see* Diabetes.

**Goitre,** cystic, C. T. Vachell, **5,** 264.
dyspnœa in, C. A. Morton, **14,** 213 ; *see also* Asthma.
exophthalmic (Graves's disease), J. M. Clarke, **5,** 17 ; F. H. Edgeworth, **14,** 41 ; W. M. Semple, **16,** 114 ; R. S. Smith, **23,** 317 ; C. E. K. Herapath, **47,** 193.
treatment by radium, A. R. Short, **47,** 185.
X-rays and anti-thyroid serum, J. M. Clarke, **25,** 201.
parenchymatous, W. A. Jackman, **46,** 277.
tachycardia with, F. H. Edgeworth, **32,** 144.
*see also* Cretinism, Myxœdema, Thyroid.

**Gout,** swellings simulating, R. L. Jones, **23,** 127 ; *and see* Neuritis, Testicle.

**Granuloma,** *see* Nasal.

**Graves's** disease, *see* Goitre.

**Griffiths,** L. M., presentation to, **18,** 97, 282.

**Growth,** *see* Cancer.

**Gullet,** foreign bodies in, E. Watson-Williams, **46,** 109.
*see also* Cancer, Deglutition, Dysphagia, Laryngoscopy.

**Gymnastics,** *see* Heart.

**Gynæcology,** evolution of, W. H. C. Newnham, **32,** 257.
and glands of internal secretion, D. C. Rayner, **48,** 1.
and surgery, E. W. H. Groves, **46,** 185.

**Hæmatemesis,** with small white kidney, T. Fisher, **22,** 243.

**Hæmaturia,** *see* Measles, Scurvy.

**Hæmophilia,** J. G. Smith, **2,** 264 ; J. E. Shaw, **15,** 240.

**Hæmoptysis,** H. H. Carleton, **45,** 39 ; *and see* Tuberculosis.

**Hæmorrhage,** accidental, J. G. Swayne, **7,** 170.
basal, with dilatation of cerebral vessels, T. M. Carter, **26,** 322.
pontine (renal changes in), J. M. Clarke, **26,** 230.
post-partum, compression of aorta, F. P. Elliott, **25,** 121, 310.
umbilical, familial, J. Taylor, **11,** 237.
*see also* Pia mater, Pregnancy.

**Hæmorrhagic** disease of new-born, W. J. H. Pinniger, **31,** 248.

**Halsted,** *see* Cancer.

**Hand,** dislocation of, T. Mitchell, **17,** 121.
significance of, A. S. Wohlmann, **14,** 117.
*see also* Deformity, Fingers.

**Harvey,** Wm., and Kingsweston, **46,** 90.
memorial, **47,** 77.

**Hay-fever,** P. Watson-Williams, **10,** 84.

**Head** injuries, *see* Cortex.

**Headache,** *see* Eye.

**Health,** King's, **45,** 319.
Ministry of, report, **37,** 178.
national, **44,** 267.
public, Royal Institute, **43,** 120.
*see also* Bristol, Milk, Preventive, Tuberculosis.

**Heart,** angina pectoris following sepsis, N. Neild, **44,** 263.
arrhythmia of, C. F. Coombs, **29,** 321.
auricular failure, C. F. Coombs and C. E. K. Herapath, **41,** 180.
fibrillation, C. E. K. Herapath, **29,** 148 ; K. H. Pridie, **45,** 125.
Stokes-Adams syndrome from digitalis in, T. F. R. Hewer, **47,** 135.
influence on percussion, D. R. Paterson, **21,** 110.
rupture, G. Thompson, **2,** 48.
sino-auricular block, H. L. Heimann, **46,** 285.
auriculo-ventricular bundle, **50,** 178.
bradycardia, R. G. Fendick and G. Parker, **17,** 113.
eye symptoms, F. R. Cross, *ibid.*
coronary obstruction, C. F. Coombs, **44,** 249 ; G. Hadfield, **44,** 257.
thrombosis, C. F. Coombs, **49,** 277.
disease, J. M. Clarke, **6,** 126.
ætiology, C. F. Coombs, **43,** 1.
alcoholic, T. Fisher, **13,** 81.
chronic, medical gymnastics in, P. E. Christofferson, **18,** 134.
congenital, J. M. Clarke, **7,** 261 ; L. Stephens, **8,** 196 ; C. B. Berry, **48,** 41.
rheumatic, in children, C. F. Coombs, **25,** 193.
geographical distribution, **44,** 140.
progress in study of, C. F. Coombs, **50,** 92.
rigid rest in, J. A. Nixon, **43,** 96.
in war, C. F. Coombs, **33,** 149.
endocarditis, septic, F. H. Edgeworth, **9,** 89.
ulcerative, J. E. Shaw, **1,** 114 ; H. L. Ormerod, **17,** 15.
examination of, posture in, W. Gordon, **42,** 224 ; *and see* Cardiograph.
failure, evidence of, P. C. Vine, **45,** 97.

**Heart**—*continued.*
lesions and mental symptoms, P. W. MacDonald, **4,** 142.
mid-diastolic murmurs, C. F. Coombs, **26,** 312.
muscle, fibroid, T. Fisher, **13,** 258.
necrosis, H. Rogers, **48,** 35.
rheumatic, T. Fisher, **18,** 16.
pain, F. G. Thompson, **27,** 193.
pericardium, adherent, in children, T. Fisher, **12,** 95.
Research Centre (Bristol), C. F. Coombs, **48,** 179.
sign in cancer, W. Gordon, **32,** 19.
Stokes-Adams syndrome, C. F. Coombs, **31,** 30 ; *and see above,* Auricular.
symptoms from dental abscess, W. R. Ackland, **41,** 79.
tachycardia, paroxysmal, P. Watson-Williams, **15,** 122 ; *see also* Goitre, Influenza.

**Hemiplegia,** œdema and loss of knee-jerk in paralysed limbs, J. M. Clarke, **17,** 97.

**Hepatic,** *see* Intestinal, Jaundice, Liver, Thrombosis.

**Heredity,** A. Prichard, **11,** 91.

**Hernia,** J. G. Smith, **8,** 1 ; C. A. Morton, **32,** 12 ; *and see* Anus.
Kocher's method in reducible, J. L. Thomas, **16,** 307.
strangulated, C. A. Morton, **14,** 23 ; N. E. F. Kemm, **48,** 151.
Richter's, C. A. Moore, **32,** 198.

**Heroin** habit, J. O. Symes, **30,** 153.

**Herpes** and brachial neuritis, G. M. Smith, **29,** 332.
zoster and varicella, R. E. Overton, **43,** 169.

**Hip,** *see* Hysterical, Tuberculous.

**History** and practice of ancient surgery, J. Swain, **27,** 289.
of *Bristol Medico-Chirurgical Journal,* P. Watson-Williams, **50,** 151.
of military medicine, G. Parker, **33,** 129.
of renal surgery and surgery of temporal bone, J. L. Firth, **41,** 1, 49.
*see also* Army, Barber-Surgeons, Bath, Boswell, Bright, Bristol, Brown, Carrick, Cholera, Dover, Elizabethes, Eye, Fletcher, Free, Gynæcology, Harvey, Homer, Infirmary, Jenner, Journal, Naval, Operation, Prichard, Progress, Shakespeare, Syphilidiator, Thot, University, Wellcome.

**Hoarseness,** B. J. Baron, **5,** 163 ; P. Watson-Williams, **19,** 203 ; E. Watson-Williams, **40,** 153 ; *and see* Laryngoscopy.
and aphonia, phthisical, B. J. Baron, **6,** 12.

**Hodgkin's** disease, E. L. Lees and F. H. Edgeworth, **30,** 150.

**Homer,** medicine in age of, F. H. Edgeworth, **34,** 105.

**Hospital** amalgamation, **36,** 92.
ball, **45,** 320.
Bristol Eye, **11,** 142.
General, **20,** 287 ; **25,** 93 ; **48,** 65.
Homœopathic, **38,** 125.
Cossham, **25,** 179, 190.
cripples', **43,** 178.
Forbes Fraser, **40,** 154.
paying, **37,** 181.
problem and Labour party, **41,** 148.
Queen Alexandra, Weston-super-Mare, **43,** 228.
Winford orthopædic, **47,** 161.
*see also* Infirmary, Tuberculosis, War.

**Hospitals,** almoner department for Bristol, **37,** 232.
Council, **47,** 339.
finance, **38,** 58.
of Madrid, E. W. H. Groves, **44,** 229.
voluntary, a public trust, **37,** 229.
or self-supporting, R. J. A. Berry, **47,** 19.

**Hotwells,** *see* Bristol ; *also* Bath, Buxton.

**Human** improvability, C. S. Myers, **49,** 31.

**Hydrocephalus,** W. B. Roué, **4,** 102.

**Hydronephrosis,** congenital dilatation of ureters with, J. M. Fortescue-Brickdale, **23,** 231.

**Hygiene,** school, J. O. Symes, **19,** 109 ; *see also* Diet, Menorrhagia.

**Hyoscine** and mydriatics, J. M. Fortescue-Brickdale, **28,** 317.

**Hypernephroma,** *see* Renal.

**Hyperpiesis,** *see* Arterial, Arteriosclerosis, Blood.

**Hyperpyrexia,** C. Elliott, **3,** 109.

**Hypnotics,** P. MacDonald, **5,** 257 ; J. M. Fortescue-Brickdale, **27,** 230 ; *and see* Insomnia.

**Hypnotism,** H. B. Costobadie, **28,** 33.
treatment of repressed memories, P. S. Connellan, **43,** 209.

**Hysterectomy,** *see* Cancer, Uterus.

**Hysterical** conditions, prolonged, **39,** 36.
disease of hips and knees, C. A. Morton, **15,** 30.
paroxysmal œdema, F. H. Edgeworth, **16,** 260.

**Hystero-epilepsy,** B. B. Fox, **14,** 224.

**Ilfracombe,** E. J. Slade-King, **8,** 257.

**Immunity,** *see* Antibodies, Sera, Tuberculin.

**Indicators,** *see* Body.

**Inebriates'** Acts, W. Cotton, **18,** 222.

**Inebriety,** A. J. H. Crespi, **7,** 244 ; J. Stewart, **23,** 144.
**Infant,** *see* Hæmorrhagic, Lac, Milk, Ophthalmia, Stomach, Syphilis.
premature, F. J. Hector, **49,** 301.
**Infantile,** *see* Paralysis, Scurvy.
**Infantilism,** E. C. Williams, **22,** 247.
**Infections,** acute ; *see* Acids, Spleen ; *also* Lymphocytosis.
focal, *see* Eyes, Nasal, Oral.
mixed, I. W. Hall, **31,** 227 ; *and see* Tuberculosis.
pyogenic, T. F. R. Hewer, **47,** 197.
**Infectious** disease in public school, W. J. Fyffe, **15,** 221.
**Infirmary,** Bristol Royal, **19,** 364, 379 ; **22,** 175 ; **25,** 272, 368 ; **38,** 122.
history of, H. Alford, **8,** 165 ; J. P. Bush, **26,** 193 ; *see also* **36,** 87.
reminiscences, A. W. Prichard, **18,** 193.
**Influence** machine, *see* X-rays.
**Influenza,** W. J. Fyffe, **8,** 147.
cyanosis in broncho-pneumonia, F. E. Fletcher, **48,** 277.
ear, nose and throat in, H. F. Mole, **26,** 226 ; A. J. M. Wright, **49,** 123.
tachycardia after, J. A. Birrell, **37,** 122.
**Insane,** general paralysis of, T. S. Sheldon, **2,** 235 ; *see also* Nerve.
**Insanity,** F. St. J. Bullen, **30,** 205 ; J. V. Blachford, **45,** 253 ; *and see* Delusions, Lunacy, Mental, War.
borderland, W. B. Kesteven, **3,** 97.
increase of, C. S. W. Cobbold, **2,** 145.
**Insomnia,** H. H. Carleton, **46,** 173 ; R. G. Gordon, **49,** 147 ; *and see* Hypnotics.
**Insulin,** *see* Diabetes.
**Intestinal** and biliary sand, G. Parker, **28,** 122.
and hepatic surgery, C. A. Morton, **15,** 317.
injury, H. F. Mole, **25,** 38.
abscess of thigh from, G. H. Lilley, **4,** 181.
intoxication, urinary products of, G. H. H. Almond, **31,** 130.
malformations, A. L. Taylor, **48,** 113 ; *and see* Meckel.
obstruction, A. Sheen, **2,** 167 ; J. G. Smith, **3,** 1 ; F. Treves, **3,** 13 ; E. L. Fox, **3,** 17 ; R. S. Smith, **3,** 20 ; N. C. Dobson, **3,** 28 ; F. R. Cross, **3,** 34 ; E. M. Skerritt, **3,** 36 ; W. M. Clarke, **3,** 38 ; H. F. A. Goodridge, **3,** 40 ; W. H. Harsant, **18,** 118 ; E. W. H. Groves, **30,** 37 ; D. P. D. Wilkie, **47,** 97 ; *and see* Cancer, Intussusception.
from appendicitis, R. Warren, **42,** 23.
from gall-stones, C. A. Morton, **41,** 126.
polypus, H. B. Runnalls, **3,** 181.
stasis, Sir W. A. Lane, **32,** 1 ; *and see* Fæces, Poisoning.
*see also* Abdominal, Appendicitis.

**Intracranial** pressure, paralysis of respiratory centre, J. L. Firth, **15,** 139 ; *see also* Papillœdema, Tumour.

**Intratracheal,** *see* Anæsthesia.

**Intubation,** *see* Larynx.

**Intussusception,** A. W. Prichard, **12,** 1 ; G. L. K. Pringle, **17,** 322 ; H. Chitty, **45,** 52.

**Iodoform,** *see* Syphilis, Thyroidism.

**Ionization** or cataphoresis, H. P. Tayler, **27,** 132 ; H. L. Jones, **27,** 138.

**Iritis,** E. R. Chambers, **48,** 51.

**Jacksonian,** *see* Epilepsy.

**Jamaica,** *see* Leprosy.

**Jaundice,** liver changes in, C. F. Coombs, **28,** 17.
surgical, C. A. Moore, **49,** 139 ; *see also* Liver.

**Jaw,** *see* Cancer, Dental.

**Jenner,** E., **15,** 70.

**Joint** disease, W. H. White, **22,** 97 ; *and see* Dislocation, Hæmophilia, Hysterical, Knee, Mobility, Osteo-arthritis, Rheumatoid, Tuberculosis.
Charcot's, *see* Ataxia.
missing links in, P. King, **21,** 137.
lesions, multiple symmetrical, C. A. Morton, **10,** 189.
swellings simulating gout and rheumatism, R. L. Jones, **23,** 127.

**Journalism,** modern medical, J. G. Smith, **2,** 217.

**Journals,** provincial medical, J. A. Nixon, **49,** 311 ; *see also* Bristol.

**Kala-azar,** E. Faraker, **43,** 183.

**Kidney,** *see* Hæmatemesis, Lindau, Renal.

**Killian,** Professor, **32,** 177.

**King's** health, **45,** 319.

**Klebs-Lœffler,** *see* Diphtheria.

**Knee-jerk,** estimation of, E. J. MacLean, **10,** 20 ; *and see* Hemiplegia.

**Knee-joint,** excision, new method, E. W. H. Groves, **25,** 88.
results of operations, A. R. Short, **30,** 52.
*see also* Hysterical.

**Koch,** *see* Tuberculosis.

**Kocher,** *see* Hernia.

**Labour,** ovarian tumour with, E. J. MacLean, **23,** 40.
premature, J. G. Swayne, **14,** 2, 126; *and see* Delivery.

**Labyrinthitis,** E. Watson-Williams, **41,** 135.

**Lac** vinum infantum, J. M. Fortescue-Brickdale, **22,** 197.

**Lachrymal** obstruction, C. B. Goulden, **26,** 143; A. E. Iles, **43,** 191.

**Lactic** acid ferments, I. W. Hall and W. A. Smith, **27,** 56.

**Lancet** centenary, **40,** 212.
commission on nursing, **49,** 97.

**Laparotomy,** *see* Abdominal, Pelvic.

**Laryngoscopy,** bronchoscopy and œsophagoscopy, direct, P. Watson-Williams, **30,** 11.

**Laryngoscope** in medicine, B. J. Baron, **4,** 145; *and see* Hoarseness.

**Larynx,** growths of, B. J. Baron, **7,** 78; *and see* Cancer.
intubation, J. Dacre, **6,** 73; H. F. Mole, **15,** 237.
*see also* Ear, Hoarseness, Tuberculosis.

**Lead** encephalopathy, J. A. James (sen.), **27,** 124.
muscular atrophy from, J. E. Shaw, **19,** 117.

**Leontiasis** ossea, E. H. E. Stack, **18,** 316.

**Leprosy** in Jamaica, W. D. Neish and T. J. Tonkin, **22,** 1.
problem and bearing on tuberculosis, Sir L. Rogers, **41,** 19.

**Leukæmia,** aleucocythæmic, R. Waterhouse, **31,** 10.
lymphatic, in child, E. C. Williams, **32,** 147.
and allied affections, J. M. Clarke, **26,** 289.
X-ray treatment, J. M. Clarke, **28,** 208.

**Library,** Bristol Medical, L. M. Griffiths, **29,** 235; C. K. Rudge, **30,** 344; *see also* **30,** 174.
bye-laws, **26,** 372.
growth of, L. M. Griffiths, **20,** 376; *also* **38,** 168; **50,** 161.
opening, **9,** 66.
removal, **29,** 192.
report, **9,** 313; **16,** 369.
things of general interest in, L. M. Griffiths, **20,** 279.

**Life** assurance examinations, R. S. Smith, **23,** 11.

**Light,** *see* Lupus.

**Lightning,** effects of, M. Critchley, **49,** 285.

**Lindau's** syndrome; association between angioma of cerebellum, polycystic pancreas and renal adenoma, G. Hadfield, **46,** 211.

**Lip,** *see* Syphilis.

**Liquids,** *see* Body.

**Lister,** R. Roxburgh, **30,** 1 ; F. R. Cross, **44,** 113.
centenary, **44,** 138.

**Liver,** acute yellow atrophy, F. H. Edgeworth, **24,** 37 ; B. M. H. Rogers, **24,** 40.
angioma of, A. L. Sheppard, **25,** 46.
changes in, with jaundice, C. F. Coombs, **28,** 17.
ruptured, L. S. Smith, **26,** 238.
*see also* Abscess, Bile, Chole-, Diverticulitis, Thrombosis.

**Locomotor,** *see* Ataxia.

**Long Fox** lecture, *see* p. 9.

**Lumbago,** F. H. Edgeworth, **14,** 304 ; C. E. S. Flemming, **24,** 110.

**Lunacy** Act 1890, **9,** 283.
practice, J. E. Shaw, **15,** 301 ; *see also* Insanity, Mental.
recommendations of Royal Commission, J. V. Blachford, **44,** 15.

**Lunatics,** unusual operations on, J. P. Bush, **17,** 219.

**Lung,** *see* Pulmonary.

**Lupus,** W. H. Harsant, **1,** 88 ; W. K. Wills, **25,** 127.
and rodent ulcer, Finsen light treatment, A. J. Harrison and W. K. Wills, **21,** 23, 119, 303.

**Lymphadenosis,** J. Meredith, **3,** 255 ; *and see* Tuberculosis.

**Lymphocytosis** of infection, T. Wilson-Smith, **45,** 187.

**Lymphosarcoma,** *see* Cancer.

**Malaria,** W. J. Fyffe, **1,** 143 ; J. O. Symes, **22,** 126.

**Malignant** disease, *see* Cancer.
pustule, L. A. Weatherly, **2,** 124.

**Malvern,** W. Tyrrell, **9,** 267.

**Mangana** water, E. L. Fox, **3,** 47.

**Manipulative,** *see* Surgery.

**Marriage,** evils of, *see* Pelvic.

**Mastoid,** *see* Ear.

**Measles,** pre-exanthematous sign, P. Watson-Williams, **18,** 139.
hæmaturia, F. G. Jenkins, **48,** 275.

**Meckel's** diverticulum, W. Sheen, **19,** 310 ; *and see* Intestinal.

**Medical** cases, operations on, E. H. E. Stack, **19,** 225, 326.
life, G. M. Smith, **20,** 289.
organization and growth of medical sciences in seventeenth century, G. Parker, **29,** 201.
teaching, *see* Bristol, Education.

**Medicine**, duty of, to the race, C. F. Walters, **47**, 1.
oriental, V. E. Green-Armytage, **28**, 40.
reports on, R. S. Smith, **9**, 184, 289.
and chemistry, F. Francis, **40**, 119.
and fine arts, J. A. Nixon, **40**, 1.
and natural sciences, J. M. Clarke, **23**, 193.
and pathology, I. W. Hall, **36**, 105.
physiology and, G. A. Buckmaster, **37**, 7.
and psychology, Sir J. H. Parsons, **39**, 1.
Royal Society, visit of Section of Anæsthetics, **48**, 159.
of Section of Surgery, **50**, 140.
and surgery, L. E. V. Every-Clayton, **37**, 193.
*see also* History, Preventive, Progress, War.

**Medulla** oblongata, tubercle of, J. M. Clarke, **7**, 194.

**Melancholia**, L. A. Weatherly, **16**, 197.

**Memories**, repressed, hypnotism for, P. S. Connellan, **43**, 209.

**Meningeal**, *see* Artery.

**Meningitis**, cerebro-spinal, J. M. Clarke, **18**, 128 ; E. H. Stack, **19**, 44 ; J. R. Charles, **31**, 142 ; in Bristol, **29**, 96 ; *and see* Septicæmia, Sinus.
suppurative, G. Thompson, **1**, 107.
operative treatment, E. Watson-Williams, **43**, 91.

**Meningocele**, J. G. Smith, **1**, 129.

**Menorrhagia** and hygienics, J. Meredith, **6**, 17, 102.

**Mental** disorder, *see* Burden, Consciousness, Heart, Lunacy, Melancholia, War.
defectives, H. L. Ormerod, **46**, 1.
health, L. G. Brock, **48**, 97.

**Mesenteric**, *see* Cyst, Thrombosis.

**Mesopotamia**, *see* War.

**Mesothelioma**, *see* Renal.

**Metabolism**, *see* Protein.

**Microscope**, unreliability of, C. H. Whiteford, **20**, 226.

**Migraine**, ocular signs, E. R. Chambers, **43**, 29.

**Military**, *see* Army, History, War.

**Milk** adulteration, E. Russell, **27**, 47 ; I. W. Hall, **27**, 48.
depots, J. M. Fortescue-Brickdale, **22**, 197 ; **23**, 132.
heating of, as it affects nutrition and health of infant, J. M. Fortescue-Brickdale, **27**, 61.
pasteurization, J. O. Symes, **18**, 275.
precautions in administering, J. M. Clarke, **27**, 57.
public health standpoint, D. S. Davies, **27**, 44.
week, **41**, 41.

**Ministry,** *see* Health.

**Mitral,** *see* Heart.

**Mobility**; sprains, dislocations and fractures (*q.v.*), J. Chiene, **21,** 97.

**Monkeys,** *see* Tumours.

**Morbus** cœrulus, P. Watson-Williams, **6,** 183.

**Morgan,** C. Lloyd, presentation to, **28,** 76.

**Morphia,** *see* Uræmia.

**Movement,** disorders of, H. H. Carleton, **48,** 135; *and see* Extremities, Mobility, Paralysis.

**Mucous** membranes, *see* Telangiectases.

**Mumps,** testicle of, F. K. Green, **1,** 229.

**Murmur,** *see* Heart.

**Muscular** atrophy, J. F. Evans, **1,** 212; C. Williams, **5,** 103; J. M. Clarke, **6,** 195.
due to lead, J. E. Shaw, **19,** 117.
neuritic, J. E. Shaw, **17,** 319.
and sclerodermia, J. A. Nixon, **21,** 328.

**Mustard-gas** poisoning, E. H. E. Stack, C. F. Coombs and R. Rolfe, **37,** 151.

**Myasthenia** gravis, J. M. Clarke, **23,** 308.

**Mydriatic,** *see* Hyoscine.

**Myocardium,** *see* Heart.

**Myo-fibroma,** myoma, *see* Uterus.

**Myxœdema,** J. M. Clarke, **7,** 258; W. A. Hayes, **12,** 230.

**Narcolepsy,** B. M. H. Rogers, **25,** 87, 144.

**Nasal** catarrh, G. F. Burder, **2,** 217; W. J. Penny, **5,** 95.
deformity, paraffin wax injections, J. P. Bush, **20,** 220; E. H. E. Stack, **27,** 119; *see also* Surgery (plastic).
excoriationes, B. J. Baron, **4,** 52.
granuloma, C. P. Crouch, **21,** 231.
respiration, defective, P. Watson-Williams, **24,** 17.
septum, operations on, P. Watson-Williams, **25,** 21.
sinuses, focal infection in, P. Watson-Williams, **42,** 121; *see also* Antrum.
frontal, suppuration, P. Watson-Williams, **33,** 24.
sphenoidal, exploration for meningococcal and other infections, P. Watson-Williams, **34,** 21.
operation, P. Watson-Williams, **34,** 157.

**Naturæ,** vis medicatrix, G. M. Smith, **27,** 321.

**Naval** volunteer, medical, in Quebec, W. K. Wills, **26,** 327.

**Neck,** *see* Cervical, Cyst, Torticollis.

**Necrosis,** *see* Cardiac, Pulmonary.

**Neoplasm,** *see* Cancer.

**Neo-salvarsan,** *see* Syphilis.

**Nephropexy,** J. L. Firth, **31,** 220 ; by carbolic acid, T. Carwardine, **23,** 24 ; *see also* Renal.

**Nephroptosis,** J. Swain, **20,** 315.

**Neuralgia,** facial, H. B. Runnalls, **3,** 249 ; from dental irritation, J. M. Ackland, **4,** 28.
trigeminal, H. H. Carleton, **49,** 47.
excision of Gasserian Ganglion, E. W. H. Groves, **26,** 234.

**Neuritis,** N. Davies, **33,** 81.
brachial, herpes and, G. M. Smith, **29,** 332.
peripheral, J. M. Clarke, **5,** 73 ; D. R. Paterson, **8,** 244 ; *and see* Muscular.
gouty, F. W. Jollye, **6,** 28.
poly-, J. M. Clarke, **33,** 9 ; B. Rogers and J. H. Bodman, **43,** 84.

**Neuroma,** cystic, J. F. Evans, **1,** 109.

**Neuroses,** *see* War.

**Nerve** degeneration, senile, resembling G.P.I., S. W. MacIlwaine, **12,** 294.
injuries, spinal and peripheral, A. W. Adams, **42,** 32.
gunshot, J. M. Clarke, **35,** 57 ; C. A. Morton, **36,** 55.
roots, posterior spinal, section of, E. W. H. Groves, **29,** 105.

**Nervous** system, autonomic, J. R. Charles, **43,** 127.
central, *see* Addison, Ataxia, Cauda, Spinal, Syringomyelia.
surgery of, P. Sargent, **42,** 101 ; *see also* Surgery (pain), Sympathectomy.

**New-born,** *see* Hæmorrhagic, Infant, Ophthalmia.

**Nitrous** oxide, *see* Ether.

**North** Pole, *see* Diet.

**Nose,** *see* Ear, Nasal.

**Nursing,** *see* Lancet.

**Obituary,** J. Alexander, **23,** 189 ; J. W. Arrowsmith, **31,** 94.
S. H. Ballance, **38,** 127 ; W. M. Barclay, **21,** 187 ; Sir B. J. Baron, **36,** 100 ; E. R. Bates, **50,** 63 ; J. Beddoe, **29,** 280, 284 ; H. A. Benham, **22,** 286 ; A. J. M. Bentley, **29,** 197 ; D. E. Bernard, **37,** 184 ; E. Blacker, **29,** 98 ; Elizabeth Blackwell, **28,** 179 ; A. G. Blomfield, **16,** 194 ; E. C. Board, **41,** 157 ; R. H. Brew, **35,** 110 ; R. Bridges, **47,** 159 ; J. Broom, **18,** 186 ; F. St. J. Bullen, **35,** 46 ; Rev. H. N. Burden, **47,** 249 ; G. F. Burder, **10,** 67 ; J. P. Bush, **47,** 342.

**Obituary**—*continued.*

C. A. Calmette, **50,** 287 ; A. Carr, **23,** 94 ; T. M. Carter, **47,** 164 ; W. F. Carter, **18,** 186 ; P. C. Chadwick, **21,** 285 ; R. H. Chilton, **20,** 191 ; C. Clarke, **42,** 66 ; J. M. Clarke, **36,** 18, 23 ; E. W. Coathupe, **43,** 238 ; R. W. Coe, **28,** 280 ; C. H. Collins, **28,** 94 ; H. W. Collins, **24,** 94 ; T. J. Coleman, **21,** 94 ; T. Coomber, **19,** 173 ; C. F. Coombs, **49,** 326 ; C. P. Coombs, **38,** 167 ; F. R. Cross, **48,** 226 ; E. Crossman, **22,** 193.

J. G. Davey, **13,** 76 ; D. S. Davies, **50,** 135 ; H. R. Dew, **29,** 287 ; N. C. Dobson, **36,** 177 ; Elizabeth W. Dunbar, **42,** 179.

W. F. B. Eadon, **30,** 285 ; R. Eager, **33,** 228 ; E. E. Eberle, **26,** 376 ; G. D. Edwards, **23,** 398 ; C. Elliott, **50,** 62 ; H. L. Evans, **42,** 151 ; U. W. Evans, **30,** 287 ; J. Evens, **34,** 201 ; H. G. Eycott, **46,** 168.

F. G. Farr, **41,** 158 ; W. Fitzpatrick, **24,** 287 ; R. Fletcher, **30,** 289 ; J. M. Fortescue-Brickdale, **38,** 63, 69 ; Sir M. Foster, **25,** 81 ; R. S. Fowler, **24,** 390 ; B. B. Fox, **20,** 150 ; C. H. Fox, **33,** 229 ; E. L. Fox, **20,** 97 ; F. Fraser, **41,** 159 ; G. B. Fraser, **24,** 286 ; A. Fry, **21,** 286 ; J. Fuller, **30,** 383 ; W. J. Fyffe, **19,** 97.

H. W. Goodden, **33,** 123 ; T. B. Goss, **28,** 379 ; A. Grace, **34,** 102 ; G. Grace, **32,** 255 ; W. G. Grace, **34,** 103 ; E. M. Grace, **29,** 197 ; A. D. Griffiths, **23,** 284 ; L. M. Griffiths, **41,** 213, **42,** 61 ; S. J. H. Griffiths, **50,** 62.

Viscount Haldane, **45,** 227 ; A. J. Harrison, **41,** 93 ; W. H. Harsant, **50,** 60 ; E. B. Hartnell, **34,** 101 ; J. P. I. Harty, **45,** 155 ; H. E. Hetling, **41,** 47 ; W. J. Hill, **35,** 136 ; E. R. Holborow, **40,** 214 ; Sir Victor Horsley, **34,** 186 ; W. R. Huggard, **29,** 382.

G. H. Irvine, **18,** 186.

H. S. Jenkins, **31,** 189 ; P. P. Johnson, **29,** 99.

B. Kendall, **19,** 381 ; H. W. Kendall, **25,** 90 ; J. G. D. Kerr, **24,** 388 ; H. A. Knight, **26,** 377 ; R. Koch, **28,** 177.

F. P. Lansdown, **35,** 42 ; R. G. P. Lansdown, **41,** 89 ; F. G. Lilley, **49,** 252.

Sir W. MacCormac, **19,** 366 ; T. A. P. Marsh, **18,** 185 ; H. Marshall, **16,** 193 ; O. C. Maurice, **30,** 181 ; H. F. Mole, **36,** 31 ; C. A. Morton, **46,** 238.

R. H. Norgate, **42,** 65.

A. Ogilvy, **32,** 189 ; E. H. Openshaw, **35,** 110 ; H. Ormerod, **21,** 189 ; H. L. Ormerod, **49,** 251 ; Sir W. Osler, **36,** 183 ; Sir Isambard Owen, **44,** 61.

J. H. Parry, **24,** 191 ; F. J. C. Parsons, **24,** 93 ; J. F. Parsons, **30,** 93 ; J. St. J. G. Parsons, **23,** 285 ; W. J. Penny, **23,** 92 ; W. J. H. Pinniger, **35,** 136 ; A. Prichard, **16,** 1 ; A. W. Prichard, **43,** 59 ; J. E. Prichard, **19,** 82 ; A. B. Prowse, **42,** 149.

**Obituary**—*continued.*
J. M. Rattray, **40,** 69 ; G. F. Rossiter, **38,** 171 ; R. Roxburgh, **35,** 35 ; C. K. Rudge, **43,** 235 ; H. T. Rudge, **21,** 190.
Sir Percy Sargent, **50,** 59 ; A. Sheen, **25,** 90 ; J. F. Shekleton, **21,** 190 ; E. J. Sheppard, **33,** 231 ; H. Skelton, **31,** 370 ; E. M. Skerritt, **25,** 97 ; F. S. Smith, **33,** 108 ; C. M. Smith, **35,** 36 ; J. G. Smith, **15,** 105 ; L. S. Smith, **38,** 186 ; R. S. Smith, **39,** 57 ; W. A. Smith, **50,** 286 ; W. H. Spencer, **28,** 276 ; E. H. E. Stack, **39,** 112 ; C. Steele, **32,** 253 ; W. H. Stevens, **31,** 287 ; E. Stock, **19,** 382 ; J. G. Swayne, **21,** 193 ; S. H. Swayne, **18,** 390 ; W. C. Swayne, **42,** 192.
G. C. Tayler, **30,** 382 ; J. Taylor, **42,** 145 ; J. T. Thompson, **29,** 195 ; H. C. Thurston, **36,** 182 ; J. Tilsley, **18,** 390 ; W. J. Tivy, **28,** 285.
E. H. Warner, **23,** 189 ; J. H. Wathen, **24,** 386 ; A. Waugh, **24,** 389 ; F. Weatherly, **28,** 283 ; T. Webster, **28,** 93 ; F. J. Wethered, **45,** 321 ; Sir George White, **34,** 194 ; J. Wilding, **19,** 173 ; R. L. Willcox, **31,** 370 ; E. Williams, **23,** 94 ; Sir Dawson Williams, **45,** 68 ; Sir George Wills, **45,** 154 ; H. H. Wills, **39,** 54 ; H. O. Wills, **29,** 287 ; Sir Vernon Wills, **48,** 65 ; R. G. Wörger, **28,** 93.

**Ocular,** *see* Eye, Rheumatoid.

**Œdema,** *see* Famine, Hemiplegia, Hysterical, Pulmonary.

**Œsophagoscopy,** *see* Laryngoscopy.

**Œsophagus,** *see* Cancer, Dysphagia, Gullet.

**Old** age, recuperative powers, H. B. Runnalls, **4,** 168.

**Olecranon,** suturing, W. P. Keall, **2,** 50.

**Omentum,** torsion of, J. Swain, **37,** 202.

**Operation** rooms, past and present, Sir W. MacCormac, **16,** 301.
*see also* Medical.

**Ophthalmia** neonatorum, prophylaxis, A. E. Iles, **44,** 125.
phlyctenular, F. R. Cross, **4,** 32.
*see also* Eye.

**Ophthalmic** reaction, opsonic, *see* Tuberculosis.

**Oral** sepsis as a source of systemic infection, W. R. Ackland, **39,** 76 ; *see also* Abscess, Dental.
surgery, N. C. Dobson, **7,** 108.

**Orbital,** *see* Aneurysm, Tumour.

**Orchitis,** *see* Testicle.

**Oriental,** *see* East, Egypt, Everest, Medicine, Mesopotamia, Plague.

**Osteo-arthritis,** paralysis of, H. Waldo, **13,** 90.

**Otitis,** *see* Ear.

**Ovarian,** *see* Cyst, Tumour.

**Ovariotomy,** T. Carwardine, **21,** 309 ; *and see* Tumour, Uterus.

**Owen,** Sir Isambard, dinner to, **38,** 169.

**Oxaluria,** *see* Phosphaturia.

**Oxycephaly,** G. H. Almond, **28,** 118.

**Oxygen,** *see* Anæmia, Drainage.

**Pain,** *see* Heart, Pelvic, Surgery.

**Palate,** *see* Cancer.

**Palsy,** *see* Paralysis.

**Pancreas,** *see* Cancer, Cyst, Lindau.

**Pancreatitis,** acute, C. A. Morton, **35,** 80.
chronic, J. M. Clarke, **30,** 25.

**Papillœdema,** with tumours, A. E. Iles, **40,** 94.

**Paraffin,** *see* Nasal.

**Paralysis** of accommodation, W. M. Beaumont, **20,** 213.
birth, F. W. Jollye.
cerebral, transient, causes of, G. Parker, **27,** 15 ; *see also* Hemiplegia.
facial, electrical reactions, J. P. I. Harty, **32,** 55.
general, *see* Insane.
infantile or anterior poliomyelitis, R. S. Smith, **2,** 175 ; R. G. Gordon, **46,** 289 ; H. Chitty, **50,** 37.
of lower limb, treatment, M. F. Brown, **45,** 295.
Landry's, A. G. Blomfield, **8,** 113.
respiratory, from pressure, J. L. Firth, **15,** 139.
of vocal cord from aneurysm, B. J. Baron, **19,** 208.
uveo-parotitic, G. Parker, **43,** 73.
*and see* Diphtheria, Extremities, Osteo-arthritis.

**Paraplegia,** J. M. Clarke, **8,** 226.

**Paratyphoid,** *see* Typhoid.

**Parkinsonian** syndrome, M. Critchley, **41,** 25.

**Parotid** swelling, C. W. Brasher, **31,** 328 ; *and see* Paralysis.

**Parturition,** *see* Delivery, Ergot, Labour, Placenta, Pregnancy, Presentation, Post-partum.

**Patella,** *see* Fracture.

**Pathological** Society meeting, **28,** 286.
specimens, C. E. S. Flemming, **20,** 222.

**Pathology,** clinical, I. W. Hall, **24,** 121.
comparative, A. J. Harrison, **12,** 273.
medicine from standpoint of, I. W. Hall, **36,** 105.

**Pediculoides,** *see* Dermatitis.

**Pellagra,** E. B. White and G. Hadfield, **44,** 31.

**Pelvic** disease (laparotomy), E. W. H. Groves, **18,** 330.
floor, relation to displacements and pain, E. W. H. Groves, **24,** 97.
inflammation from rectal diverticula, W. C. Swayne, **35,** 91; *and see* Diverticulitis.
organs, unsound; evils of marriage and pregnancy in women with, A. E. A. Lawrence, **14,** 219.

**Peptic** ulcer, T. Carwardine, **40,** 71; *see also* Duodenum, Stomach.

**Percussion,** *see* Cancer, Heart, Pulmonary.

**Pericardium,** adherent, in children, T. Fisher, **12,** 95.

**Pericolitis,** T. Carwardine, **31,** 333.

**Peritonitis,** C. H. Whiteford, **26,** 53; F. Fraser, **40,** 29.

**Pernicious,** *see* Anæmia.

**Personality** (review), C. Lloyd Morgan, **43,** 104.

**Pharmacopœia,** British, O. C. M. Davis, **32,** 292.
copyright of, **49,** 168.

**Pharmacy** and pharmaceutical chemistry, progress of, O. C. M. Davis, **23,** 120.

**Pharyngeal** pouch of Rathke, T. Carwardine, **15,** 210.

**Pharyngitis,** P. Watson-Williams, **7,** 189; *see also* Tuberculosis.

**Phosphaturia** and oxaluria, alternating, F. H. Edgeworth, **31,** 18; C. W. J. Brasher, **31,** 21.

**Phthisis,** *see* Tuberculosis.

**Physiology** and medicine, C. A. Buckmaster, **37,** 7.

**Physique** of boys, B. M. H. Rogers, **27,** 27.

**Physicians,** Association of, **41,** 153.

**Pia** mater, minute hæmorrhages, T. S. Sheldon, **1,** 225.

**Pick's** disease, H. J. Orr-Ewing, **38,** 90.

**Pigmentation,** *see* Amenorrhœa.

**Placenta** prævia, J. G. Swayne, **1,** 166.

**Plague,** oriental, E. Klein, **20,** 108.
in Bristol 1916, D. S. Davies, **35,** 16; *and see* Diphtheria.

**Plastic,** *see* Surgery.

**Pleural** effusions, A. Sheen, **2,** 14; *see also* Empyæma.

**Pleurisy,** initial signs, F. H. Edgeworth, **21,** 36.
serous, due to pneumococci and tubercle bacilli, F. H. Edgeworth, **31,** 146.

**Pneumococci** in urine, G. M. Smith, **13,** 115.

**Pneumonia,** A. Fells, **27,** 336; *and see* Influenza, Pulmonary.
cerebral symptoms in children, F. H. Edgeworth, **31,** 308.
lobar, causes of death, F. H. Edgeworth, **37,** 88.
unusual complications, J. M. Clarke, **25,** 89, 108.

**Pneumothorax,** artificial, in phthisis, H. Chitty, **30,** 141.
personal experience, E. Mariette, **30,** 189.

**Poisoning,** self-, C. J. Whitby, **14,** 307.
*see also* Blood, Bromoform, Cocaine, Duboisia, Lead, Malaria, Mustard-gas, Rhus, Tea, Toxicity.

**Poliomyelitis,** *see* Paralysis.

**Polycythæmia,** *see* Erythræmia.

**Polyneuritis,** *see* Neuritis.

**Pons,** hæmorrhage, renal changes in, J. M. Clarke, **26,** 230.
*see also* Tumour.

**Postgraduate,** *see* Education.

**Post-partum** complications, A. E. A. Lawrence, **8,** 73 ; *and see* Hæmorrhage.

**Posture,** J. K. Spender, **1,** 184.
in examination of heart, W. Gordon, **42,** 224.

**Pregnancy,** albuminuria in, R. S. S. Statham, **49,** 219 ; J. A. Nixon, **49,** 229 ; *see also* Eclampsia.
extra-uterine, A. E. A. Lawrence, **10,** 73 ; A. E. A. Lawrence and C. A. Morton, **11,** 29 ; R. S. S. Statham and H. L. Shepherd, **48,** 15.
tubal, W. H. C. Newnham, **15,** 152 ; H. F. Mole, **21,** 39 ; E. W. H. Groves, **22,** 46 ; C. H. Whiteford, **24,** 126.
hæmorrhages of, A. E. A. Lawrence, **19,** 196.
operations on uterus, etc., during, W. C. Swayne, **30,** 31.
and parturition, cerebral lesions, W. C. Swayne, **25,** 209.
vomiting of, J. Meredith, **4,** 95.
*see also* Pelvic, Tumour.

**Premature,** *see* Infant, Labour.

**Presentation** of axilla, F. P. Elliott, **19,** 130.
occipital, J. G. Swayne, **16,** 108.

**President** in Canada, **44,** 274.

**Presidential** address, *see* p. 5.

**Preventive** medicine, modern, D. S. Davies, **18,** 289 ; **24,** 193.
Institute of, Bristol University, **50,** 267.

**Priapism,** A. H. Boys, **1,** 224.

**Prichard,** J. C., **50,** 169.
and Bristol Medical Library Society, **30,** 174.

**Prognosis,** P. H. Pye-Smith, **25,** 1.

**Progress** and practice, R. Roxburgh, **16,** 285.
Bristol's contribution to medical, **50,** 165.
in dermatology, W. K. Wills, **45,** 1.
in ear, nose and throat disease, B. J. Baron, **19,** 289.

**Progress**—*continued.*
in treatment of empyema, J. A. Nixon, **38,** 82.
medical, F. R. Cross, **9,** 241.
in pharmacy, O. C. M. Davis, **23,** 120.
in rheumatic heart disease, C. F. Coombs, **50,** 93.
in surgery, F. P. Lansdown, **4,** 217 ; H. P. Symonds, **13,** 161 ; F. Lace, **36,** 1 ; A. R. Short, **46,** 243.
*see also* Cancer, Delivery (forceps), Gynæcology.

**Prolapse** and cystocele, W. C. Swayne, **37,** 81.
rectal, D. Robertson and D. G. C. Tasker, **46,** 71.

**Prostate,** enlarged, C. W. J. Brasher, **14,** 150 ; A. R. Short, **50,** 23.

**Proteins** and protein metabolism, J. M. Fortescue-Brickdale, **24,** 318.

**Psychology,** R. Eager, **22,** 289 ; Sir J. H. Parsons, **39,** 1.
Medico-psychological Association meeting, **45,** 70.

**Psychotherapy,** R. G. Gordon, **38,** 11 ; *and see* Hypnotism.

**Ptosis,** W. M. Beaumont, **20,** 131.

**Public** health, *see* Bristol, Milk, Preventive, Sanitary, Tuberculosis.

**Puerperium,** H. J. D. Smythe, **46,** 59 ; *see also* Cretinism, Eclampsia, Tumour.

**Pulmonary** congestion, relieved by aspiration of aorta, J. Dacre, **3,** 189.
disease, H. H. Carleton, **42,** 211 ; **43,** 54.
bacteriology of suppurations complicating, J. O. Symes, **17,** 30.
with Friedländer's bacillus, J. M. Clarke, **32,** 1.
injections, R. S. Smith, **3,** 164.
localized necrosis, J. M. Clarke, **20,** 122.
œdema ; acute suffocative, J. A. Birrell, **44,** 239.
paroxysmal, C. E. S. Flemming, **31,** 121.
percussion notes, G. H. H. Almond, **27,** 215.
*see also* Influenza, Pneumonia, Silicosis, Tuberculosis.

**Pyæmia,** portal, *see* Diverticulitis.

**Pyelography,** *see* Renal.

**Pylorectomy,** subsequent history of case, A. W. Prichard, **24,** 53.

**Pylorus,** congenital hypertrophic stenosis, T. Fisher and N. Neild, **22,** 123.
*see also* Cancer.

**Pyocyaneus,** *see* Septicæmia.

**Pyogenic** infections, T. F. R. Hewer, **47,** 197.

**Pyrexia,** W. H. Spencer, **3,** 217 ; *see also* Hyperpyrexia, Temperature.

**Quackery,** *see* Eye.

**Quebec,** *see* Naval.

**Radiography,** *see* X-rays.
**Radiotherapy,** W. K. Wills, **22,** 39 ; E. Dore, **45,** 85 ; J. Taylor, **24,** 289 ; *and see* Goitre, Leukæmia, Lupus.
**Radium,** therapeutic use, J. M. Davidson, **28,** 1 ; A. E. H. Pinch, **41,** 97 ; H. S. Souttar, **46,** 19 ; Sylvia B. Wigoder, **50,** 233.
for goitre, A. R. Short, **47,** 82.
**Rathke,** pouch of, T. Carwardine, **15,** 310.
**Raynaud's** disease, W. J. Penny, **6,** 48 ; H. Waldo, **6,** 272 ; D. A. Alexander, **30,** 247.
**Recklinghausen's** disease, L. P. Ashton, **47,** 219.
**Rectal,** *see* Pelvic, Prolapse.
**Reminiscences** and a forecast, Sir E. J. MacLean, **48,** 83 ; *and see* Army.
**Renal** adenoma, *see* Lindau.
calculi, E. J. T. Jones, **21,** 245 ; *see also* X-rays.
changes with hæmorrhage into pons, J. M. Clarke, **26,** 230.
disease, D. G. C. Tasker, **43,** 161 ; *see also* Hæmatemesis, History, Hydronephrosis, Nephro-.
pyelography in, A. W. Adams, **46,** 49.
efficiency, C. Clarke, **39,** 13 ; J. O. Symes, **39,** 25 ; G. Hadfield, **39,** 28.
hypernephroma or mesothelioma, J. Swain, **31,** 213.
**Research,** ethics of, E. de L. O'Leary, **31,** 242.
**Respiration,** *see* Nasal, Paralysis.
**Respirators,** M. Mœller, **2,** 136.
**Retinoscopy,** A. B. Prowse, **1,** 200.
**Rheumatic** disease, conference at Bath, **45,** 70.
*see also* Heart, Progress, Testicle.
**Rheumatism,** acute ; throat as source of infection, P. Watson-Williams, **22,** 215.
histology of aortic wall, J. J. J. Giraldi, **46,** 145.
and chorea, J. J. S. Lucas, **22,** 239.
latent, C. M. Jessop, **5,** 153.
*see also* Joint.
**Rheumatoid** arthritis, vasomotor and ocular phenomena, R. L. Jones, **20,** 328.
bacillus of, A. S. Wohlmann **14,** 123.
**Rhinitis,** *see* Hay-fever, Nasal.
**Rhus** venata poisoning, A. W. Prichard, **9,** 22.
**Rib,** cervical, R. Waterhouse, **31,** 232.
**Richter,** *see* Hernia.
**Röntgen,** *see* X-rays.
**Rodent,** *see* Lupus.
**Rötheln,** W. J. Fyffe, **10,** 1 ; J. Meredith, **11,** 171.
**Round** the world, R. Roxburgh, **21,** 202.

**Saints,** diseases bearing the names of, R. Fletcher, **30,** 295.

**Sanatorium,** *see* Tuberculosis.

**Sanitary** Institute, Royal, meeting, 1906, **24,** 178, 270.

**Sarcoma,** *see* Cancer.

**Scarlet** fever, G. M. Smith, **2,** 263 ; J. Wallace, **42,** 157.
secondary rashes, J. O. Symes, **15,** 20.

**School** hygiene, J. O. Symes, **29,** 109 ; *see also* Boys, Infectious, Surgery.

**Sclerodermia** and muscular atrophy, J. A. Nixon, **21,** 328.

**Sclerosis,** *see* Disseminated, Spinal.

**Scurvy,** infantile, B. M. H. Rogers, **21,** 318 ; C. Elliott, **21,** 323.
causing hæmaturia, R. S. Smith, **21,** 320.

**Sea,** *see* Therapeutics.

**Secretin** for diabetes, J. R. Charles, **24,** 218.

**Sepsis,** *see* Angina, Dental, Nasal, Oral, War.

**Septicæmia,** meningococcal, C. B. Perry, **45,** 207.
pyocyaneus, J. M. Clarke, **31,** 4.

**Sera** and vaccines, J. M. Fortescue-Brickdale, **24,** 1.

**Serositis** of unusual distribution, H. J. Orr-Ewing, **38,** 90.

**Sewer,** diseases of men who worked in, J. M. Fortescue-Brickdale, **24,** 1.

**Shakespeare** and the medical sciences, L. M. Griffiths, **5,** 225.

**Shoulder-centre,** *see* Cancer.

**Siddall,** J. B., **30,** 84.

**Siepmann** prize, **38,** 123.

**Sight,** *see* Vision.

**Silicosis,** J. A. Nixon, **48,** 253 ; G. B. Bush, **48,** 259.

**Sinus,** *see* Nasal, Thrombosis.

**Sino-,** *see* Auricular.

**Skiagraphy,** *see* X-rays.

**Skin,** *see* Dermatitis, etc., Telangiectases.

**Skull,** development, E. Fawcett, **28,** 97.
localization of foramina, E. Fawcett, **13,** 173.

**Sleep,** *see* Hypnotic, Insomnia, Somnambulism.

**Small-pox** in Bristol, D. S. Davies, **27,** 97.
variola nigra, D. S. Davies, **20,** 49.

**Smith,** J. G., **50,** 178; memorial, **16,** 379.

**Smith,** R. S., dinner, **31,** 1 ; resignation as Editor, **30,** 369, 377.
**Soap,** antiseptic and disinfectant properties, J. O. Symes, **17,** 193.
**Somnambulism,** J. M. Clarke, **12,** 81.
**Specialism** and medical curriculum, P. Watson-Williams, **34,** 21.
**Spermatic** cord, torsion, J. L. Firth, **22,** 320.
**Sphenoidal,** *see* Nasal.
**Sphygmograms,** copying, W. M. Semple, **16,** 312 ; *see also* Cardiograph.
**Spina** bifida, E. L. Fox, **2,** 45 ; C. A. Morton, **10,** 14.
**Spinal** cases, J. M. Clarke, **6,** 120 ; *see also* Anæsthesia, Cancer, Cauda Equina, Nerve, Tuberculosis.
sclerosis, J. E. Shaw, **2,** 270.
**Spleen,** in acute pyogenic infection, T. F. R. Hewer, **47,** 197.
enlargement, E. L. Fox, **3,** 145.
*see also* Cancer, Erythræmia, Splenomegaly.
**Splenectomy,** J. Swain, **28,** 223.
for splenomegaly, J. A. Nixon, **31,** 325 ; E. W. H. Groves, **31,** 331.
**Splenomegaly** (Banti's), J. M. Clarke, **21,** 14 ; E. C. Williams, **24,** 42 ; **25,** 43, 88 ; R. Roxburgh, **25,** 286.
*see also* Abdominal.
**Sprain,** *see* Mobility.
**Sprue,** C. Begg, **23,** 138.
**Stammering,** *see* War.
**Sterilization** of unfit, H. C. Bristowe, **48,** 189.
**Sterility,** clinical, R. S. S. Statham, **44,** 41.
**Stokes-Adams** syndrome, *see* Heart.
**Stomach,** dilatation, J. M. Clarke, **7,** 21 ; **13,** 181.
disease, J. M. Clarke, **9,** 73, 280 ; *see also* Cancer, Pylorus.
suicidal perforation, L. Stephens, **6,** 113.
ulcer, origin of, W. Gordon, **19,** 100.
perforated, C. A. Morton, **26,** 207.
surgical aid in, J. A. Nixon, **26,** 217.
*see also* Gastroenterostomy, Peptic.
and duodenum, end results of operation, A. R. Short, **29,** 220 ; *and see* Pylorectomy.
ulcer of, in Infants, J. A. Nixon, **45,** 72.
**Stone,** *see* Calculus.
**Stools,** examination of, G. H. H. Almond, **33,** 95.
**Stricture,** *see* Cancer, Urethra.
**Structure** and function, relationship, J. R. Charles, **49,** 1.
**Styracol,** C. Reinhardt, **25,** 54.

**Subungual** exostoses, W. R. Williams, **22,** 17.

**Subdiaphragmatic** abscess, J. Harper, **2,** 184.

**Sugar** tolerance tests, H. D. Pyke, **45,** 129.

**Suicide,** attempted (Clifton Suspension Bridge), J. F. Evans, **3,** 124 ; *see also* Stomach.

**Sulphonal** in epilepsy, G. A. Bannatyne, **9,** 261.

**Suppuration,** *see* Abscess, Ear, Pulmonary, Spleen.

**Suprarenal** virilism, E. Fawcett, G. Hadfield and P. Phillips, **43,** 20 ; J. R. Charles, **49,** 115 ; J. A. Birrell, **49,** 119.

**Surgery,** C. F. Walters, **41,** 202.
anæsthesia and, A. L. Flemming, **48,** 233.
in France, J. Swain, **34,** 128.
and gynæcology, borderland, E. W. H. Groves, **46,** 185.
insulin in, J. A. Nixon, **43,** 199.
intestinal and hepatic, C. A. Morton, **15,** 317.
manipulative, A. G. T. Fisher, **45,** 165.
medicine and, L. E. V. Every-Clayton, **37,** 193.
oral, N. C. Dobson, **7,** 108.
for pain, E. R. Carling, **50,** 81 ; *see also* Neuralgia.
plastic, H. D. Gillies, **43,** 122 ; *see also* Nasal.
school, A. W. Prichard, **13,** 241.
*see also* Abdominal, Cancer, History, Intestinal, Nervous, Progress, War.

**Surgical** notes, W. H. Harsant, **1,** 82 ; T. Carwardine, **26,** 317.

**Surprises** in my career, J. P. Bush, **21,** 289.

**Symonds,** J. A., and cholera in Bristol, **50,** 57.

**Sympathectomy,** periarterial, E. W. H. Groves, **45,** 273.

**Symphysiotomy,** W. C. Swayne, **17,** 11.

**Synovitis,** *see* Tuberculosis.

**Syphilidiator,** an old-time, H. J. Norman, **28,** 334.

**Syphilis,** H. Waldo, **25,** 289.
(chancre) on lip, W. H. Harsant, **1,** 97 ; of lip and gum, W. J. Penny, **6,** 46.
cerebro-spinal, J. M. Clarke, **29,** 1.
congenital, W. H. Harsant, **1,** 83.
constitutional, W. J. Penny, **6,** 45.
diagnosis, A. M. Critchley, **46,** 127.
household, and acquired syphilis in infants, J. A. Nixon, **29,** 301.
iodoform for, R. S. Smith, **1,** 128.
neosalvarsan for, J. A. Nixon, **31,** 24.
V.D. treatment, L.G.B. scheme, **34,** 188.
and tuberculosis, P. Watson-Williams, **2,** 155.

**Syringomyelia,** F. H. Edgeworth, **12,** 100.

**Tabes,** *see* Ataxia.

**Tachycardia,** *see* Heart, Goitre, Influenza.

**Talipes,** *see* Club-foot.

**Tea** poisoning, N. Neild, **29,** 312.

**Teeth,** effect of general conditions on, W. R. Ackland, **34,** 118.
*see also* Dental, Oral.

**Teething** myth, R. C. Clarke, **38,** 28.

**Telangiectases,** familial multiple, of skin and mucous membranes, G. R. Scarff, **50,** 113.

**Temperature** in apoplexy, J. M. Clarke, **17,** 97.

**Temporal** bone, *see* Ear, History (surgery).

**Tenby,** J. G. Lock, **10,** 97.

**Testicle,** misplaced, W. H. Harsant, **1,** 96.
gouty, F. K. Green, **1,** 227.
of mumps, F. K. Green, **1,** 229.
rheumatic, W. J. Penny, **6,** 49.

**Tetanus,** epidemic, A. Sheen, **1,** 173.

**Tetany,** *see* Thyroid.

**Therapeutics** of sea voyage, R. Roxburgh, **21,** 1.
notes, J. M. Fortescue-Brickdale, **31,** 279, 373 ; **32,** 88, 181, 249.

**Thermal** procedures, C. J. Whitby, **18,** 1.

**Thomsen's** disease, W. H. Spencer, **2,** 159.

**Thot,** an exalted surgeon and physician, F. Bushnell, **16,** 123.

**Throat,** *see* Aphonia, Ear, Hoarseness, Larynx, etc., Pharyngitis, Progress, Rheumatism, Tuberculosis.

**Thrombosis,** coronary, C. F. Coombs, **49,** 277 ; *and see* Coronary.
embolism and, A. W. Owen, **47,** 29.
of femoral artery, A. E. Barker, **2,** 84.
of hepatic veins, with ascites, T. Fisher, **20,** 209.
mesenteric, G. M. Smith, **24,** 216.
portal, idiopathic, B. M. H. Rogers, **17,** 107.
of lateral sinus, C. F. Pickering, **9,** 155 ; J. L. Firth, **19,** 36 ; J. P. I. Harty, **38,** 73.

**Thyroid,** surgery of, W. H. Harsant, **6,** 265 ; *and see* Goitre.
followed by tetany, D. Wood, **48,** 205.

**Thyroidism** and iodoform, A. R. Short, **28,** 122.

**Toe-nail,** ingrowing, J. G. Smith, **2,** 99.

**Tongue** disease, W. J. Penny, **6,** 37.
outgrowths, B. J. Baron, **8,** 80 ; *and see* Cancer.

**Tonsil** enucleation, E. W. H. Groves, **23,** 32.
and adenoid operations, unsuccessful, A. J. M. Wright, **40,** 84.
*see also* Cancer, Tuberculosis.

**Torquay,** P. Q. Karbeek, **11,** 93.

**Torsion,** *see* Omentum, Spermatic.

**Torticollis,** spasmodic, R. S. Smith, **14,** 297.

**Tropics,** *see* Blood, Leprosy, Medicine, Oriental.

**Trypanosomiasis,** J. O. Symes, **21,** 325.

**Tubal,** *see* Abortion, Cyst, Pregnancy.

**Tubercle** in monkeys, tumour and, W. R. Williams, **25,** 149.

**Tuberculin** and immunity, J. W. Taylor, **30,** 338 ; *and see* Sera, Tuberculosis.
test in cattle, G. S. Pollard, **19,** 210.

**Tuberculosis,** abdominal, J. Swain, **22,** 31.
of colon, E. W. H. Groves, **27,** 203.
of elbow, W. H. Harsant, **1,** 19.
relation to erythema nodosum, J. O. Symes, **45,** 233.
of hip-joint in childhood, C. A. Morton, **22,** 207.
of larynx, C. Elliott, **2,** 260 ; B. J. Baron, **6,** 12 ; P. Watson-Williams, **9,** 27.
tuberculin for, P. Watson-Williams, **32,** 136.
bearing of leprosy problem on, Sir L. Rogers, **41,** 19.
of lymph-nodes of lung, L. R. Jordan, **47,** 225.
of medulla oblongata, J. M. Clarke, **7,** 194.
miliary, J. M. Clarke, **15,** 148.
and natural selection, D. S. Davies, **30,** 135.
of nose and pharynx, A. J. M. Wright, **30,** 45 ; G. R. Scarff, **45,** 73.
officer, L. J. Short, **31,** 153.
ophthalmic reaction, Calmette's, J. M. Fortescue-Brickdale, **26,** 112.
pulmonary (consumption, phthisis), R. S. Smith, **6,** 185 ; R. Roxburgh, **30,** 193.
contagious, R. S. Smith, **1,** 1 ; **6,** 225 ; evidence against, E. M. Skerritt, **1,** 48.
diagnosis, C. Muthu, **26,** 131 ; L. J. Short, **35,** 1.
hæmoptysis (*q.v.*) ; treatment, N. Neild, **26,** 124.
Koch's treatment, E. M. Skerritt and B. J. Baron, F. J. Wethered, **8,** appendix.
notification, voluntary, D. S. Davies, **23,** 351.
opsonic treatment, J. M. H. Munro, **26,** 97.
pneumothorax, artificial, H. Chitty, **30,** 141.
personal experience, E. Mariette, **30,** 189.
prognosis, J. J. S. Lucas, **24,** 12.
relation to public health, D. S. Davies, **37,** 211.

**Tuberculosis**—*continued.*
pulmonary—
resistance, acquired, S. L. Cummins, **40,** 41.
sanatoria for, L. A. Weatherly, **18,** 305; C. Braine-Hartnell, **19,** 2.
Frenchay Park, **38,** 124.
Winsley, **22,** 79; **28,** 191.
foundation, **21,** 170; report (first annual), **24,** 278.
management, **32,** 179, 247.
statistics (and other preventable disease), W. H. Symons, **19,** 7.
sudden death from asphyxia, W. B. Roué, **2,** 181.
tuberculin for mixed infections, N. Bardswell, **32,** 125.
vaccines and sera, J. M. Fortescue-Brickdale, **24,** 1.
for secondary infections, S. H. Habershon, **32,** 97.
spinal, treatment, H. H. Tomkins, **3,** 184; A. R. Short, **37,** 19.
in ventral position, K. H. Pridie, **50,** 261.
and syphilis, P. Watson-Williams, **2,** 155.

**Tumour,** bladder, cystotomy, J. P. Bush, **13,** 8.
brain, J. Taylor, **2,** 128.
and papillœdema, A. E. Iles, **40,** 94.
buccal, J. Swain, **13,** 94.
cerebellar, J. M. Clarke, **24,** 97.
ovarian, J. M. Lawrie, **10,** 186.
complicating pregnancy, labour and puerperium, E. J. MacLean, **23,** 40.
pelvic, obstructing labour: Cæsarean section, W. C. Swayne, **22,** 120.
of pons, P. Watson-Williams, **9,** 163.
ponto-cerebellar, A. W. Adams, **49,** 309.
of thigh, fatty, J. G. Smith, **1,** 126.
and tubercle in monkeys, W. R. Williams, **25,** 149.
*see also* Cancer, Ovariotomy, Renal, Uterus.

**Typhlitis,** J. G. Swayne, **4,** 108; *and see* Appendicitis.

**Typhoid** carrier, vesical calculus in, D. Wood, **37,** 148.
fever and typhoid carriers, D. S. Davies and I. W. Hall, **26,** 39.
with bacillus coli infection, J. I. Noble, **48,** 323.
in isolation hospital in France, J. M. Fortescue-Brickdale, **36,** 72.
treatment, J. M. Clarke, **16,** 15; F. P. Elliott, **18,** 225.
ulcer, perforated, H. G. Kyle, **21,** 30; (paratyphoid B.), F. H. Bodman and P. Bodman, **47,** 317.

**Ulcer,** *see* Duodenum, Peptic, Stomach, Typhoid; Rodent, *see* Lupus.

**Umbilical** cord, compression of, J. G. Swayne, **2,** 229.
hæmorrhage, familial, J. Taylor, **2,** 237.

**University,** Bristol, **20,** 76 ; **24,** 76, 171, 372 ; **25,** 78 ; **26,** 173, 192 ; **28,** 74, 363, 380 ; **31,** 96 ; **38,** 118.
medical faculty, **42,** 138.
new buildings, **42,** 73, 139 ; *and see* Bristol (Medical School), Preventive, Wills.
College, Bristol, anniversary, **44,** 58.
Medical School association with, F. R. Cross, **44,** 74.
incorporation with, **11,** 266 ; **25,** 93.
of South-West, **42,** 188.
Strasbourg, **43,** 121.

**Urachus,** patent, W. J. Penny, **6,** 36.

**Uræmia,** morphia in, T. M. Carter and F. H. Edgeworth, **20,** 231.

**Ureter,** *see* Calculus, Cystoscopy, Hydronephrosis, X-rays.

**Urethra,** caruncle of, A. E. A. Lawrence, **18,** 23.
stone in, C. A. Morton, **28,** 127 ; *see* Calculus.
stricture, J. G. Smith, **2,** 154 ; E. W. H. Groves, **28,** 325.

**Urinary** affections, urotropin in, J. O. Symes, **20,** 27.
products of intestinal intoxication, G. H. H. Almond, **31,** 130.
tract, *see* Bacillus Coli.

**Urine,** *see* Eclampsia, Phosphaturia, Pneumococci, Pregnancy.
retention, aspirator, W. Fairbanks, **4,** 242.

**Urticaria,** factitious, H. Waldo, **8,** 29 ; A. J. Harrison, **8,** 32.

**Uterus,** appendages of, conservative surgery, J. Swain, **21,** 211 ; *see also* Pelvic.
removal of, J. G. Smith, **4,** 1, 73 ; *and see* Ovariotomy, Pregnancy (tubal).
erosion of cervix, G. Hadfield, **43,** 151 ; H. J. D. Smythe, **43,** 156.
fibroid, W. H. C. Newnham, **32,** 37.
fibroids of, W. C. Swayne, **18,** 123.
myo-fibroma of, J. Swain, **22,** 22.
myoma of, W. R. Williams, **17,** 1.
neoplasms of, W. R. Williams, **17,** 314 ; *see also* Cancer, Endometrioma.
operations on, during pregnancy, W. C. Swayne, **30,** 31.

**Uveo-parotitic** paralysis, G. Parker, **43,** 73.

**Vaccines,** *see* Tuberculosis.

**Varicella,** *see* Herpes.

**Variola,** *see* Small-pox.

**Vasomotor** phenomena, *see* Rheumatoid.

**Vein,** *see* Thrombosis.

**Venereal,** *see* Syphilis.

**Venesection,** *see* Eclampsia.

**Vertebræ,** cervical, inflammation round, J. K. Spender, **11,** 1.

**Vertigo,** E. Watson-Williams, **44,** 119.

**Vessels,** injury to main, in civil practice, R. V. Cooke, **47,** 311.
cerebral, dilatation, T. M. Carter, **26,** 322.

**Victoria,** Queen, in memoriam, **19,** 1.
Jubilee Convalescent Home, **19,** 167.

**Virilism,** *see* Suprarenal.

**Viscera,** abdominal, displacement, T. Fisher, **19,** 217.

**Vision,** colour, Edridge-Green test, **28,** 175.
evolution of sense of, F. R. Cross, **33,** 163 ; *see also* Eye.
field of, defects in, F. R. Cross, **29,** 33.

**Vocal** cord, paralysis (aneurysm), B. J. Baron, **19,** 208 ; *and see* Aphonia, Hoarseness, Laryngoscopy.

**Volunteer,** *see* Naval.

**Vomiting** in children, J. Swain, **24,** 116.
*see also* Anæsthesia, Pregnancy.

**War,** the Great, and Bristol profession, **32,** 242, 315.
bullets and other metallic foreign bodies, removal of, R. G. P. Lansdown, **33,** 157.
cardiac disease, C. F. Coombs, **33,** 149.
in Egypt, General Hospital, E. W. H. Groves, **34,** 11.
in France, enteric diseases seen in isolation hospital, J. M. Fortescue-Brickdale, **34,** 72.
experiences of ear, nose and throat specialist, J. P. I. Harty, **34,** 39.
surgery, J. Swain, **34,** 128.
X-rays, W. Cotton, **34,** 144.
effect on mental conditions in Bristol, R. H. Norgate, **37,** 95.
in Mesopotamia, disease, F. P. Mackie, **36,** 118.
medicine and surgery, C. F. Coombs, **34,** 136.
surgery in Indian General Hospital, P. S. Connellan, **35,** 113.
neuroses of, J. M. Clarke, **34,** 49.
-œdema, a food-deficiency disease, J. A. Nixon, **37,** 137.
stammering in, R. G. Gordon, **36,** 143.
women doctors with army, H. B. Hanson, **33,** 88.
wounds, Carrel-Dakin method of preventing sepsis, J. Swain, **35,** 68.
reduction of disabilities from, W. Gordon, **34,** 1.
*see also* Ambulance, Army, Eye, History, Mustard-gas, Naval, Nerve.

**Warts,** B. G. Pullin, **5,** 269.

**Watson-Williams,** P., resignation of editorship, **43,** 119.

**Wellcome** Historical Medical Museum, **43,** 229 ; S. H. Daukes, **46,** 236.

**Weston-super-Mare,** C. V. Hitchins, **7,** 198.

**Wills,** Sir George, **43,** 48, 227.

**Wills,** Henry Herbert, physics laboratory, **44,** 275.

**Women,** *see* Pelvic, War.

**Wounds,** *see* Eye, Finger, Nerve, Vessels, War.

**Wright,** J., & Sons, centenary, **43,** 52.

**X-ray(s)** camera, W. Cotton, **29,** 118.
centroscope and episcope, W. Cotton, **32,** 202.
diagnosis of calculi, J. Swain, **15,** 1 ; J. Taylor, **20,** 44.
of needle in foot, C. H. Whitby, **14,** 238.
of silicosis, G. B. Bush, **48,** 259.
history of a fracture, J. Taylor, **18,** 214.
in France, W. Cotton, **34,** 144.
influence machine for, W. Cotton, **17,** 222.
pictures, J. Taylor, **18,** 214.
estimation of simple enlargement by means of visible shadows, W. Cotton, **27,** 226.
proportional representation and comparison, W. Cotton, **25,** 326.
right and wrong side of, W. Cotton, **30,** 227.
stereoscopic, W. Cotton, **20,** 218 ; **23,** 216.
*see also* Radiotherapy.

**Zoological** Gardens, Clifton, observations in, A. J. Harrison, **31,** 318.

**Zymotic** enteritis, *see* Typhoid.

