# Addendum

**Published:** 1933

**Authors:** 


ADDENDUM.

Page 59. **Paralysis :** birth, F. W. Jollye, *add* **7**, 87.

